# Iced photochemical reduction to synthesize atomically dispersed metals by suppressing nanocrystal growth

**DOI:** 10.1038/s41467-017-01521-4

**Published:** 2017-11-14

**Authors:** Hehe Wei, Kai Huang, Da Wang, Ruoyu Zhang, Binghui Ge, Jingyuan Ma, Bo Wen, Shuai Zhang, Qunyang Li, Ming Lei, Cheng Zhang, Joshua Irawan, Li-Min Liu, Hui Wu

**Affiliations:** 1 State Key Laboratory of New Ceramics and Fine Processing, School of Materials Science and Engineering, Tsinghua University, 100084 Beijing, China; 2 Beijing Computational Science Research Center, 100193 Beijing, China; 3 Beijing National Laboratory for Condensed Matter Physics, Institute of Physics, Chinese Academy of Sciences, 100190 Beijing, China; 4 Shanghai Synchrotron Radiation Facility, Shanghai Institute of Applied Physics, Chinese Academy of Sciences, 201204 Shanghai, China; 5 AML, CNMM, Department of Engineering Mechanics, State Key Laboratory of Tribology, Tsinghua University, 100084 Beijing, China; 6 State Key Laboratory of Information Photonics and Optical Communications and School of Science, Beijing University of Posts and Telecommunications, 100876 Beijing, China; 7 School of Materials Science and Engineering, Shanghai Institute of Technology, 201418 Shanghai, China; 8 School of Material Science and Engineering, University of New South Wales, Sydney, NSW 2052 Australia; 9 School of Physics and Nuclear Energy Engineering, Beihang University, Beijing, 100191 China

## Abstract

Photochemical solution-phase reactions have been widely applied for the syntheses of nanocrystals. In particular, tuning of the nucleation and growth of solids has been a major area of focus. Here we demonstrate a facile approach to generate atomically dispersed platinum via photochemical reduction of frozen chloroplatinic acid solution using ultraviolet light. Using this iced-photochemical reduction, the aggregation of atoms is prevented, and single atoms are successfully stabilized. The platinum atoms are deposited on various substrates, including mesoporous carbon, graphene, carbon nanotubes, titanium dioxide nanoparticles, and zinc oxide nanowires. The atomically dispersed platinum on mesoporous carbon exhibits efficient catalytic activity for the electrochemical hydrogen evolution reaction, with an overpotential of only 65 mV at a current density of 100 mA cm^−2^ and long-time durability (>10 h), superior to state-of-the-art platinum/carbon. This iced-photochemical reduction may be extended to other single atoms, for example gold and silver, as demonstrated in this study.

## Abstract (web summary)

Photochemical synthesis is a popular approach to fabricate metallic nanoparticles, however stabilizing individually-dispersed atoms by this method remains challenging. Here, the authors freeze their precursor solution prior to UV irradiation to obtain atomically-dispersed platinum catalysts with high electrocatalytic performance.

## Introduction

Atomically dispersed metals have become an area of growing scientific interest due to their unique chemical and physical properties, as well as their applications as high performance catalysts^1–6^. The synthesis of metal single atoms by traditional solution chemistry methods is extremely difficult mainly because of the diffusion, aggregation and nucleation of the product atoms in the liquid phase. In fact, for over a century, in-solution chemical and photochemical reduction of metal ions has been studied as one of the most common methodologies for the synthesis of metal nanocrystals^7–14^. Controlling the morphology and size of these nanocrystals has relied strongly on the fundamental understanding of the nucleation and growth of solids during liquid phase reactions. In typical liquid phase chemical/photochemical reductions, it is recognized that nucleation represents the very first step that atomic or molecular species must undergo before emerging as nanocrystals; generally, a precursor is reduced or decomposed to generate atoms, followed by their evolution into nuclei and then nanocrystals^15, 16^. Recently, using in situ electron microscopy, Mirsaidov et al.^17^ demonstrated that the reduction of metal ions in an aqueous solution results in nanocrystals in three major steps: spinodal decomposition into solute-rich and solute-poor liquid phases, nucleation of amorphous nanoclusters within the metal-rich liquid phase, and crystallization of these amorphous clusters^17^. It has been recognized that to generate sub-nanometer clusters or even single atoms, the diffusion of atoms and the formation of metal nuclei should be prevented.

Recently, a number of strategies have been proposed for downsizing solution processed nanocrystals to sub-nanometer clusters or even single atoms. Confining the chemical/photochemical reduction process to a nanoscale space can greatly influence the nucleation of materials. Sub-nanometer clusters or single atoms can be obtained in the confined pores of zeolite^18^, mesoporous carbon, metal organic frameworks (MOFs), and so on, using solution synthesis routes such as the impregnation method^19, 20^ and the organometallic complex method^21, 22^. Despite significant progress, the demonstration of facile and high-yield synthesis of atomically dispersed metals with high density and high stability remains challenging. It is an interesting and open question whether, we can effectively prevent the nucleation and crystal growth in solution synthesis. For example, photochemical reduction chemistry, which involves the absorption of photons and electronically excited states^23, 24^, has been widely applied for the syntheses of nanomaterials^25, 26^, especially for nanocrystals of noble metals, including gold, silver, platinum, palladium, and so on^27, 28^. In this process, the nanocrystal formation and growth strongly rely on the diffusion of the ions and atoms inside the liquid environment. Considering this fact, we propose to freeze the precursor solution for the photochemical reaction. Instead of directly growing nanocrystals in the liquid phase, the ice lattice naturally confines the dispersed reactants and therefore possibly restricts the photochemical reduction products, preventing nucleus formation.

Herein, we experimentally demonstrate that atomically dispersed Pt can be effectively synthesized via iced-photochemical reduction using ultraviolet (UV) irradiation of frozen chloroplatinic acid (H_2_PtCl_6_) aqueous solution. Pt single atoms (Pt_1_) can be stabilized on various substrates, including amorphous carbon film, mesoporous carbon (MC), graphene, multi-walled carbon nanotubes (MWCNTs), TiO_2_ nanoparticles, and ZnO nanowires. The Pt_1_/MC material is an active and stable electrocatalyst for the hydrogen evolution reaction (HER), with significantly improved performance compared with state-of-the-art commercial platinum/carbon (Pt/C) catalysts.

## Results

### Concept and process of iced-chemistry

**Fig. 1:**
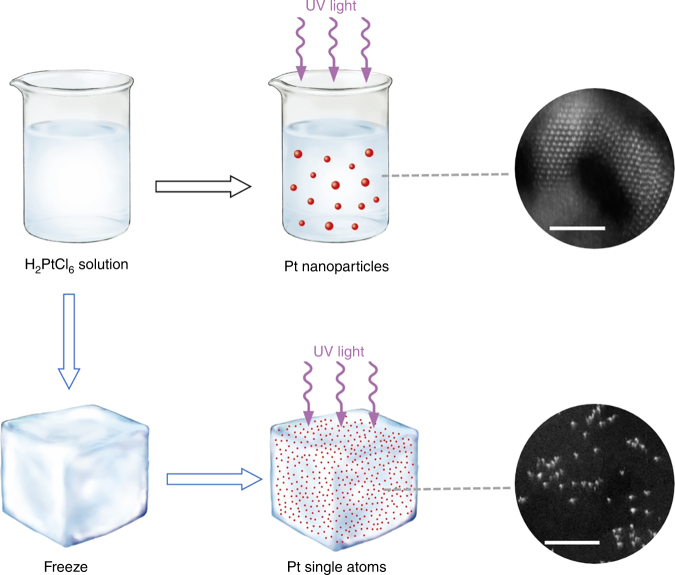
Schematic illustration the iced-photochemical process. In a conventional photochemical reduction of H_2_PtCl_6_ aqueous solution, Pt nanocrystals are formed by the agglomeration and nucleation of Pt atoms (top row). Conversely, in the bottom row, we froze the H_2_PtCl_6_ solution before exposing it to UV irradiation and obtained Pt single atoms dispersed in ice. The ice lattice naturally confined the ions/atoms and prevented their nucleation. (scale bar, 2 nm)

**Fig. 2:**
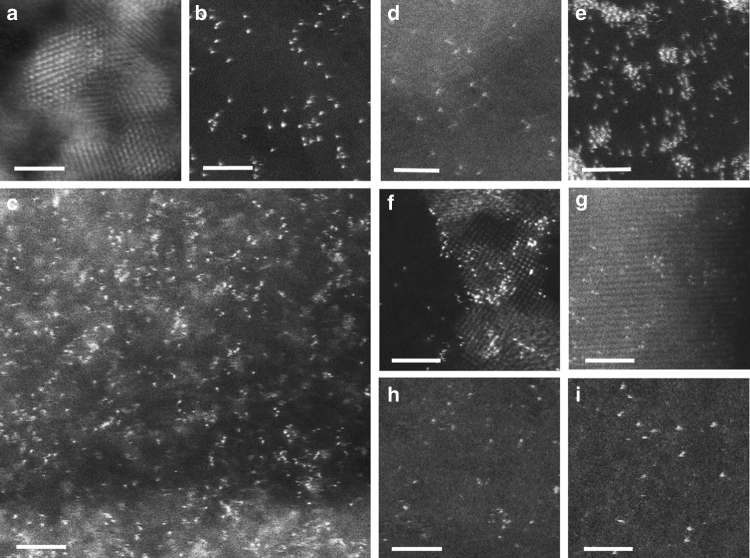
HAADF-STEM images of Pt single atoms. **a** Pt nanocrystals with a size of ~2 nm formed by normal photochemical reduction of H_2_PtCl_6_ aqueous solution. **b** Pt single atoms dispersed on ultrathin carbon film, with each bright dot corresponding to one individual Pt atom, with a size of ~0.1 nm. **c** Densely and homogeneously dispersed Pt single atoms on mesoporous carbon. **d** Pt_1_/MWCNTs, indicating that Pt existed completely as isolated single atoms. **e** Pt_1_/graphene, with concomitant Pt single atoms, nanoclusters and sub-nanometer clusters. **f** Atomically dispersed Pt on titanium oxide nanoparticles. **g** Pt single atoms attached on the surface of zinc oxide nanowires. **h** Ag and **i** Au single atoms prepared by a similar iced-photochemical route. (scale bar, 2 nm)

**Fig. 3:**
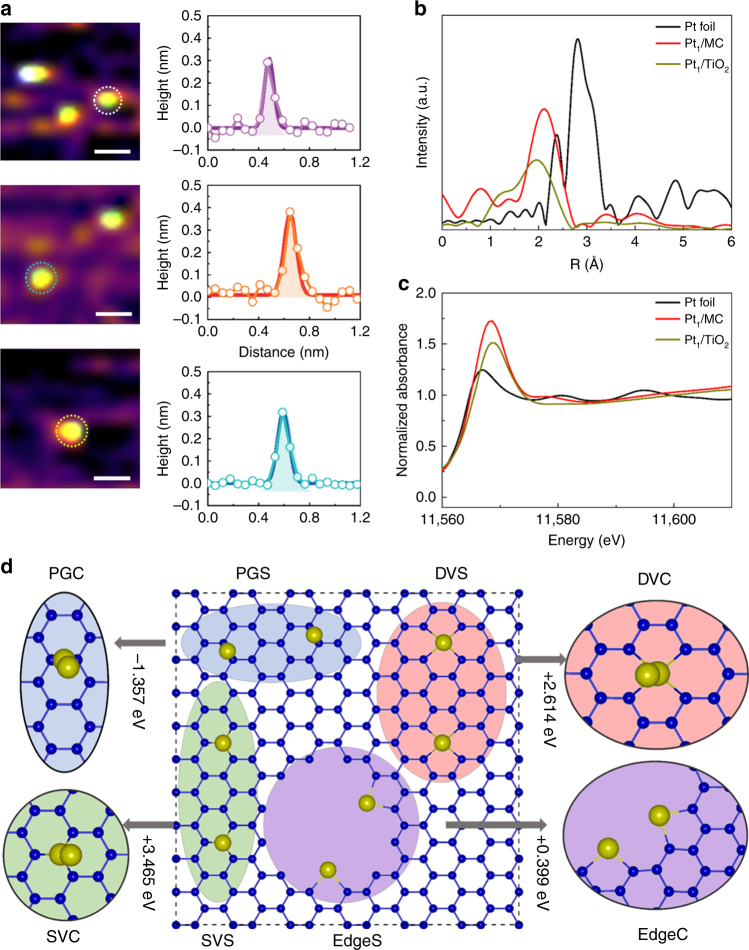
Characterizations and structure of Pt single atoms. **a** Typical STM images of Pt single atoms dispersed on ultrathin carbon films (constant current mode). **b** EXAFS spectra of bulk Pt foil and Pt single atoms absorbed on TiO_2_ and MC. **c** Normalized XANE structure spectra at the Pt L_3_-edge. **d** Structure and size distribution for Pt single atoms and Pt clusters on mesoporous carbon. The Pt single atom and clustering configurations are denoted by XS and XC, respectively, where X indicates the Pt atoms adsorbed on different defects (SVs and DVs) and edges. The yellow and blue balls represent the Pt and C atoms, respectively. (scale bar, 0.2 nm)

The iced-photochemical synthesis of Pt single atoms is illustrated in Fig. 1. First, H_2_PtCl_6_ aqueous solution was rapidly frozen by dipping the container in liquid nitrogen, with a cooling rate of 40 °C min^−1^ (Supplementary Fig. 1). Benefiting from the ultralow temperature of liquid nitrogen, H_2_PtCl_6_ aqueous solution can be frozen very quickly to yield an ice layer with a homogeneous concentration of Pt precursor (Supplementary Fig. 2). Note that all chemicals, the solution and the ice were kept in dark conditions to avoid possible H_2_PtCl_6_ decomposition by ambient light. Second, the frozen solution was exposed to UV irradiation on a cooling stage with a temperature of −25 °C for 1 h to ensure the photochemical reduction of Pt (IV). After the UV treatments, the frozen solution was kept overnight in dark conditions at room temperature to yield a clear aqueous solution. The solution was subsequently mixed with various dispersions containing MC, graphene, MWCNTs, TiO_2_ nanoparticles, and ZnO nanowires. After centrifuging, washing and drying the samples multiple times, the products from the iced-photochemical process were stabilized on these substrates. We characterized the products with aberration-corrected high-angle annular dark field-scanning transmission electron microscopy (HAADF-STEM). Obviously, the traditional UV irradiation of H_2_PtCl_6_ aqueous solutions (as shown in the upper line of Fig. 1) produced Pt crystals with the size of a few nanometers (Fig. 2a). In comparison, the iced-photochemical reduction (lower line of Fig. 1) leads to high density areas of isolated Pt atoms (Fig. 2b, Supplementary Fig. 3). The atoms were identified as platinum, confirmed by electron energy loss spectra (EELS, Supplementary Fig. 4). No Pt nanoclusters and nanoparticles were observed, as shown in the TEM images and selected area electron diffraction (SAED) spectra (Supplementary Fig. 4d). The size distribution of Pt single atoms (Supplementary Fig. 5) showed a significant majority of isolated atoms with a size of ~0.1 nm (Supplementary Fig. 6) on ultrathin carbon film. When the concentration of H_2_PtCl_6_ solution reached 7.3 mM, the Pt single atoms partially aggregated under the same experimental conditions using the iced-photochemical reduction (Supplementary Fig. 7). The STEM images in Fig. 2c–g, Supplementary Fig. 8 display the Pt atoms attached on various substrates, including MC, MWCNTs, graphene, TiO_2_ nanoparticles, and ZnO nanowires. Atomically dispersed Pt can be observed on most substrates with the exception of graphene, where partially aggregated clusters have been found. The synthesized materials were further characterized with scanning tunneling microscopy (STM), X-ray absorption fine structure (XAFS), and X-ray diffraction (XRD). Figure 3a shows the typical STM images of atomically dispersed Pt on ultrathin carbon films. With atomic resolution, Pt single atoms appeared as single-peak protrusions ~0.2 nm in diameter and ~0.3 nm in height, in good agreement with previous literature^29^. For the XAFS study, the X-ray absorption near edge structure (XANES) of Pt single atom materials (Pt_1_/MC and Pt_1_/TiO_2_) exhibited increased white-line intensity compared to that of the Pt foil, in accordance with previous reports^1, 5^ (Fig. 3b). As shown in Fig. 3c and Supplementary Table 1, there was a notable peak ~2.1 Å from the Pt-C/O contribution for Pt_1_/MC and ~2.0 Å from the Pt-O contribution for Pt_1_/TiO_2_ and no peak ~2.76 Å from the Pt-Pt contribution, confirming the isolated states of Pt single atoms on the mesoporous carbon. This was consistent with the direct observation of the atomic dispersion of Pt by STEM (Fig. 2). The binding energies of Pt 4f_7/2_ and 4f_5/2_ for Pt_1_/MC (Supplementary Fig. 9) were 72.3 eV and 75.1 eV (corresponding to Pt^δ+^). The results were in agreement with previous reports^3, 5^. XRD results further confirmed the absence of a Pt lattice, as shown in Supplementary Figs. 10, 11.

### Synthesis mechanism

The above experimental results demonstrated that Pt single atoms were fabricated on substrates, indicating that it is possible that the Pt single atoms could have been stabilized in water. To comprehend this, several different sizes of Pt cluster, such as Pt single atom, Pt dimer, and Pt_13_, were explored in water at both room temperature and 1000 K using first-principles molecular dynamics (FPMD) (Supplementary Fig. 12). For the Pt single atom system, two Pt single atoms were initially placed into water to check how they behaved in aqueous solution. The results showed that the two Pt single atoms do not form a Pt dimer in aqueous solution at 300 K for 100 ps (Supplementary Fig. 12a), while the Pt single atom automatically formed the H-Pt-OH species through dissociating water molecules during FPMD at 1000 K. To further check the oxidation state of Pt in H-Pt-OH, the electronic structure of H-Pt-OH in water was analyzed using the form of Pt-H_2_O as a reference (Supplementary Fig. 13, Supplementary Table 2). In the case of the Pt single atom in Pt-H_2_O, the calculated Bader charge of the Pt atom was −0.094 *e*; thus, no obvious charge transfer occurred between Pt and water. Meanwhile, as shown in Supplementary Table 3, only one of five Pt-d electron orbitals (d_z_^2^) were nearly half-filled (1.377 *e*). In this case, the valence electron configuration of Pt in Pt-H_2_O was 5d^9^6s^1^, suggesting the approximately neutral oxidation state for Pt in Pt-H_2_O. When the H-Pt-OH structure was formed, one of five Pt-d orbitals (d_*x*_^2^_−*y*_^2^) became nearly half-filled (1.377 *e* by partial charge analysis), and two Pt-d orbitals (d_*xy*_/d_*xz*_) became partially filled with 1.796 and 1.809 *e*. These two orbitals interacted with hydroxyl and protons, respectively. Meanwhile, the Bader charge of Pt in H-Pt-OH was 0.408 *e*. It should be noted that the absolute oxidation state (OS) is difficult to determine by a computational method^30^. Here, only the relative oxidation state can be determined based on the Bader charge and *d*-orbital^31^. Considering that both d_*xy*_ and d_*xz*_ were partially occupied (not half-filled), the corresponding Pt state in H-Pt-OH was between approximately +1 and +2. Correspondingly, the Bader charge results showed that one of the H in H-Pt-OH changed to −0.048 *e*, which was reduced to the zero state.

Meanwhile, we initially started with the Pt(OH)_2_^2−^ in water system to check whether Pt(OH)_2_ instead of H-Pt-OH could be formed in the real situation during the fabrication of Pt single atoms. If Pt(OH)_2_ could be formed in the Pt-water system, the water should also be dissociated into hydroxyl and protons. Thus, the Pt(OH)_2_ was simulated along with two extra protons (Supplementary Fig. 12c). While the two hydrogen protons were initially located away from Pt(OH)_2_, the whole system remained electrically neutral. The Pt(OH)_2_ could spontaneously transform into H-Pt-OH along with the extra protons during MD simulations, in which one of the OH^-^ in Pt(OH)_2_ reacted with H^+^ to form H_2_O, and the other hydrogen proton in the water bound to Pt, as seen from the intermediate and final states in Supplementary Fig. 12c. This confirmed that H-Pt-OH was the stable species for Pt single atoms in aqueous solution instead of Pt(OH)_2_. As for both Pt_2_ and Pt_13_, water dissociation also occurred on them, forming H-Pt_2_-OH or H-Pt_13_-OH like species, while either Pt_2_ or Pt_13_ itself remained intact and did not show any tendency to form any isolated Pt, such as H-Pt-OH. This result clearly indicated that it is vital to inhibit the formation of the Pt cluster at the initial step to retain the Pt in the form of single atoms, and no nucleation between two H-Pt-OH occurs in our 100 ps FPMD simulations. More interestingly, as shown in Supplementary Fig. 14, the H and OH in H-Pt-OH structure can be easily released as water on MC, leaving the Pt single atom on MC with the release of approximately −0.345 and −1.105 eV at the single vacancy (SV) and double vacancy (DV) defect sites, respectively. This process further suggested that the Pt single atom in solution is an essential step for the fabrication of Pt_1_/MC.

Another important question is whether the Pt single atoms can be stabilized on different surfaces. As mentioned above, atomically dispersed Pt on the surface of amorphous carbon, MC, MWCNTs, TiO_2_, and ZnO was successfully fabricated, while both Pt single atoms and clusters were found on graphene. For this purpose, the energy differences (*ΔE*_*c*_) between the isolated Pt atom and the Pt dimer were calculated for graphene, MC and TiO_2_. Here, negative values mean clustering is most stable, and vice versa. First, considering the large negative value of *ΔE*_*c*_ (−1.357 eV) for Pt_1_/graphene, it was reasonable to expect the agglomeration of Pt single atoms on the graphene surface. Moreover, the graphene in our work was prepared by physical methods and contained few defective sites. There was almost no charge transfer occurring between Pt atoms and the perfect graphene to stabilize Pt atoms. Therefore, Pt atoms on perfect graphene were present as clusters and single atoms (Fig. 3e). Second, MC usually contains all kinds of defects, such as SV and DV, or edges. As shown in Supplementary Fig. 15, Supplementary Table 4, Pt single atoms preferred to adsorb on different defects, such as SVs, DVs or edges in MC. The isolated Pt atoms were more stable by at least ~0.4 eV than the corresponding Pt dimers located at one defect and an edge, as shown in Fig. 3d. Thus, Pt atoms energetically favoured the single-atom configuration rather than clustering on MC at defects/edges, while the Pt single atoms clustered on the pristine graphene, as observed in our STEM experiments (Fig. 2).

Next, let us discuss how the Pt single atoms anchor on the metal oxides. Here, we took anatase TiO_2_ as an example. It is well-known that the oxygen vacancies in the anatase TiO_2_ (101) exist in the subsurface layer rather than on the surface^32–34^. Interestingly, our results showed that when the Pt single atoms adsorbed on the surface, the oxygen vacancy spontaneously migrated from the subsurface to the surface, leaving a vacancy site for Pt adsorption. The adsorption energy per Pt atom was −4.10 eV at the oxygen vacancy, which was smaller than the one (−2.19 eV) on the perfect site of TiO_2_. Meanwhile, the isolated Pt atom on each vacancy was more stable by 0.91 eV than the formation of one dimer on the vacancy (Supplementary Fig. 16). Thus, the Pt atoms preferred to form isolated atoms on each O_v_ rather than clustering on one O_v_ site of TiO_2_ (101). Furthermore, based on the successful synthesis of Pt single atoms, we proposed that the iced-photochemical method could be further developed as a more generalized strategy to fabricate other materials. We processed the AgNO_3_ and H_2_AuCl_4_ solution with a similar iced-photochemical reduction and obtained Ag and Au single atoms homogeneously dispersed on ultrathin carbon film (Fig. 2h, i, Supplementary Figs. 17−18).

### Electrocatalyst performance

**Fig. 4:**
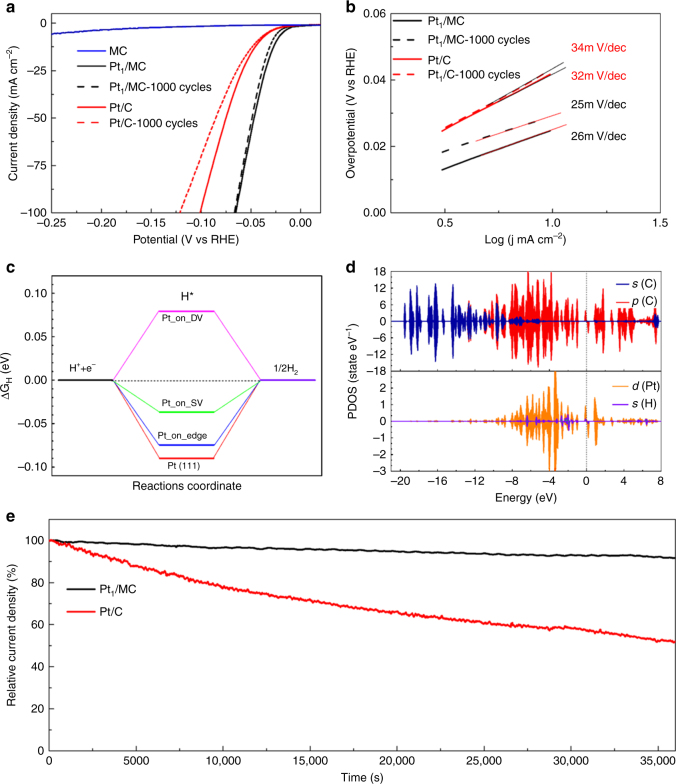
HER activity of Pt_1_/MC and commercial Pt/C in 0.5 M H_2_SO_4_. **a** Polarization curves of Pt_1_/MC and Pt/C before and after 1000 CV cycles. **b** Tafel plots of Pt_1_/MC and Pt/C (collected before and after 1000 CV cycles). **c** Reaction mechanism for the HER, calculating *ΔG*_H*_ for atomic H adsorption on different defects/edge on mesoporous carbon. **d** Partial density of states (PDOS) of the Pt adsorbed on a single vacancy system after H adsorption. The Fermi level is shifted to zero. **e** Long-time running of Pt_1_/MC and Pt/C electrocatalysts at the same overpotential of 30 mV

Downsizing Pt metals to single atoms certainly provided an effective approach to maximizing the atom efficiency for their catalytic applications^1–6^. Electrocatalysis of the hydrogen evolution reaction (HER), which has been widely used as a fundamental important process for water splitting and hydrogen production, was chosen as a model reaction. A Pt_1_/MC (Supplementary Fig. 19) electrode was connected to a typical three-electrode setup in 0.5 M H_2_SO_4_ electrolyte at room temperature to evaluate the catalytic performance. Our results showed that the Pt_1_/MC catalysts exhibited a comparable HER activity to that of commercial Pt/C catalysts at the same Pt loading of 10 µg cm^−2^ (Fig. 4a, Supplementary Fig. 20). The overpotential at a current density of 100 mA cm^−2^ (equal to a mass activity of 10 A mg^−1^) for Pt_1_/MC was 65 mV (Supplementary Fig. 21, Supplementary Fig. 22), with a promising decrease of more than 30 mV compared to the commercially available Pt/C catalyst as well as other single atom Pt-based catalysts (Supplementary Table 5). Importantly, Pt_1_/MC presented a negligible overpotential increase, while commercial Pt/C catalysts showed a 22 mV overpotential increase after 1000 cyclic voltammetry (CV) sweeps. Actually, we have measured the HER polarization curves for the reactivated Pt/C sample after stability measurements for ten hours at an overpotential of 30 mV, which exhibited an enlarged overpotential at the current density of 100 mA cm^−2^ (Supplementary Fig. 23). This suggested that the deceased activity of Pt/C can be attributed to irreversible detachment and/or agglomeration of the Pt nanoparticles in the Pt/C sample, which is in line with previous reports^35, 36^. By contrast, Pt_1_/MC showed a negligible overpotential increase after 1000 cyclic voltammetry sweeps at the same conditions, attributed to the strong interaction between the isolated Pt atoms and MC. As shown in the STEM image of Pt_1_/MC after 1000 CV cycles (Supplementary Fig. 24), almost no clusters can be observed with a similar density of Pt single atoms on mesoporous carbon. Moreover, the mass activity of Pt_1_/MC and Pt/C is shown in Supplementary Fig. 25, in which the mass activity of Pt_1_/MC was more than three times greater relative to that of Pt/C from 40 mV to 60 mV overpotential, especially for the Pt_1_/MC sample after the stability measurement of 1000 CV cycles. To provide further insight into the HER mechanisms on Pt_1_/MC and Pt/C catalysts, Fig. 4b shows that the Tafel plots of Pt_1_/MC and Pt/C catalysts were ~30 mV dec^−1^, consistent with the previous reports and the known mechanism of the HER on Pt-based HER catalysts^35^. The relatively smaller Tafel slopes of Pt_1_/MC, derived from both the initial HER polarization curve and the one after 1000 CV cycles, strongly indicated the superior HER performances of Pt_1_/MC. As discussed above, these results suggested the significantly enhanced HER specific activity of atomically dispersed platinum on mesoporous carbon at a lower consumption of precious Pt. To further assess the long-term stability of Pt_1_/MC and Pt/C catalysts as another important issue in the development of advanced electrocatalysts, chronopotentiometric measurements at a fixed overpotential of 30 mV over extended periods as long as 10 h are presented in Fig. 4e, showing that there was a very small decay for Pt_1_/MC and an obvious decline for Pt/C (8.4% vs. 48.0%). This sharp contrast indicated that the significant stability of Pt_1_/MC catalysts could be ascribed to a relatively stronger interaction between the Pt single atoms and mesoporous carbon substrates.

## Discussion

It is important to understand why Pt_1_/MC exhibits a more active HER performance compared to commercial Pt/C catalyst. For this purpose, the Gibbs free energies for atomic H adsorption, *ΔG*_H*_, on different MC defects and edges were systematically considered and compared to pure Pt (111). As shown in Fig. 4c, Supplementary Fig. 26, the Pt single atom on MC can adsorb more than one hydrogen atom^35^; thus, the hydrogen production on Pt single atoms grown on MC can follow the Tafel mechanism $(2{\rm H} ^ \ast \rightarrow {\rm H}_2)$. Interestingly, a smaller negative $\Delta G_{{H} ^\ast}$ was observed for all Pt single atoms adsorbed on the MC with a SV (Pt_SV_), a DV (Pt_DV_,) and an edge (Pt_Edge_). The calculated $\Delta G_{{H} ^\ast}$ values for H adsorbed on Pt_SV_, Pt_DV_ and Pt_Edge_ sites were −0.049, 0.080, and −0.075 eV, respectively. The $\Delta G_{{H} ^\ast}$ for the Pt single atom systems became smaller than the corresponding value for commercial Pt/C, confirming the enhancement of HER activities in Pt single atoms loaded on MC.

The HER activity mainly originates from the unique electronic properties of Pt single atoms on MC with respect to the conventional Pt surfaces. When Pt atoms are bound to MC, charge transfer occurs between MC and Pt^37^. For Pt_1_/MC, the discrete 5d-orbitals of the Pt single atoms were mixed with the C 2p orbitals around the Fermi level for Pt_SV_ (Supplementary Fig. 28). The single Pt atom was positively charged (+0.270 *e*), and the C atom obtained the electron based on Bader charge analysis (Supplementary Table 6). In this case, the Pt single atoms on the SV contained unoccupied 5d densities of states, which is vital for the HER^38, 39^. Upon H chemisorption on Pt_1_/MC (Fig. 4d), the 5d orbitals of the Pt single atoms interacted strongly with the 1 s orbital of the H atoms, leading to electron pairing and formation of hydride. In addition, more unoccupied Pt (5d) states were found above the Fermi level, which was consistent with the calculated charge transfer from the Pt atoms to the H atoms, as shown in Supplementary Table 6. This unique change in the electronic properties of the Pt single atoms on MC should be the primary reason for the increased HER activity of Pt single atoms on MC.

In conclusion, we have developed an iced-photochemical process to synthesize atomically dispersed Pt catalysts. Compared to commercial Pt/C catalysts, the Pt single atoms loaded on mesoporous carbon achieved superior catalytic activity as well as improved stability for HER electrocatalysis. The iced-chemistry route not only provides a promising pathway for green synthesis of materials including single atoms and sub-nanometer clusters, but also opens up possibilities for tuning the solid nucleation and growth of wet-chemistry reactions. We propose that numerous conventional solution-phase chemical/photochemical reactions can use our iced-chemistry reactions to achieve the production of novel materials.

## Methods

### General information

Except when otherwise noted, all chemicals were purchased and used without purification. Additional details of materials and characterization techniques used are presented in Supplementary Methods.

### Synthesis of Pt_1_/MC

A 0.3 mg ml^−1^ H_2_PtCl_6_ solution was prepared using chloroplatinic acid and deionized water. A 1.5 ml H_2_PtCl_6_ solution was frozen quickly and entirely using liquid nitrogen. Then, the H_2_PtCl_6_ ice was irradiated by a UV lamp for 1 h; meanwhile, the environment was kept at −25 °C in a lyophilizer (LGJ-10N), avoiding the melting of ice. The power density of the UV light near ice was measured to be 0.89 mW cm^−2^ using a radiometer (PL-MW 2000). After UV irradiation, the H_2_PtCl_6_ ice was melted at room temperature. All of these treatments were performed in a dark environment except for the UV irradiation treatment. Therefore, a Pt single atom solution was prepared. The mixed solution was composed of a 10 ml 5 mg ml^−1^ mesoporous carbon solution and a 25 ml 0.3 mg ml^−1^ Pt single-atom solution and was filtered. The mesoporous carbon was synthesized using a facile aqueous route^40, 41^. Then, the mixed samples were cleaned entirely using deionized water to remove the unreacted H_2_PtCl_6_. The Pt_1_/MC samples were dried naturally at room temperature.

### XAFS experiment and data processing

XAFS measurements at the Pt L_3_-edge in both transmission (for Pt foil) and fluorescence (for samples) mode were performed at BL14W1^42^ in the Shanghai Synchrotron Radiation Facility (SSRF). The electron beam energy was 3.5 GeV, and the stored current was 260 mA (top-up). A 38-pole wiggler with a maximum magnetic field of 1.2 T inserted in the straight section of the storage ring was used. XAFS data were collected using a fixed-exit double-crystal Si (111) monochromator. A Lytle detector was used to collect the fluorescence signal, and the energy was calibrated using a Pt foil. The photon flux at the sample position was 2.1 × 10^12^ photons per second.

The raw data analysis was performed by using the IFEFFIT software package according to the standard data analysis procedures^43^. The spectra were calibrated and averaged, the pre-edge background was subtracted, and the spectra were post-edge normalized using the Athena program in the IFEFFIT software package. The Fourier transformation of the *k* ^3^-weighted EXAFS oscillations, *k* ^3^ *·*χ(*k*), from *k* space to R space was performed over the range of 3.0–11.5 Å^−1^ (3.0–14.2 for Pt foil) to obtain a radial distribution function. Data fitting was performed by the Artemis program in IFEFFIT.

### Electrocatalytic measurements

The catalyst dispersion or ink was prepared by mixing commercial Pt/C (20 wt%) or as-prepared Pt_1_/MC (2.6 wt%, confirmed by an ICP-MS test) in a 2 ml mixture of 1.0 ml isopropyl alcohol, 0.95 ml water and a 0.05 ml 5 wt% Nafion solution followed by ultrasonication for 30 min. For Pt/C, after dispersing 2 mg of commercial Pt/C in 2 ml of mixture solution, the concentration of Pt element in Pt/C was 0.2 mg ml^−1^. Then, 9.8 ml of the ink was uniformly loaded onto the freshly polished glassy carbon electrodes (diameter = 5 mm), and the corresponding loading of Pt amounts was 0.01 mg cm^−2^ (10 µg cm^−2^). For Pt_1_/MC, 37.7 ml of the ink (0.052 mg ml^−1 ^Pt element concentration after 4 mg Pt_1_/MC was dispersed in 2 ml mixture solution) was also uniformly loaded onto the same freshly polished glassy carbon electrodes, and the corresponding loading of Pt amounts was also 0.01 mg cm^−2^.

All electrochemical tests were performed in a conventional three-electrode electrochemical cell using an Autolab potentiostat (PGSTAT-302N) equipped with the Nova 1.11 software. A rotating-disk glassy-carbon electrode or an l-form glassy-carbon electrode was used as the working electrode, while a carbon rod electrode and Ag/AgCl (with saturated KCl as the filling solution) were used as the counter and reference electrodes, respectively. All potentials measured were calibrated to the reversible hydrogen electrode (RHE) using the following equation: $E_{\rm RHE} = E_{{\rm Ag}/{\rm AgCl}} + 0.197 + 0.059^ \ast {\rm pH}$. To accelerate the diffusion of the H_2_ gas bubbles formed at the catalyst surface during the hydrogen evolution reaction (HER), the polarization curves were recorded using a rotating-disk electrode with 90% IR compensation at the rotation rate of 1600 r.p.m. A total of 1000 cyclic voltammetry sweeps were carried out at the voltage range from +0.3 to −0.1 V (vs. RHE) with a scan rate of 100 mV s^−1^ in 0.5 M H_2_SO_4_ to test the stability of Pt_1_/MC and Pt/C catalysts; meanwhile the stability measurements were also conducted using an l-form electrode at the overpotential of 30 mV.

### DFT calculations

Our calculations were performed based on density functional theory (DFT) calculations, as implemented in the Vienna ab initio package (VASP)^44, 45^. The general gradient approximation of Perdew−Burke−Ernzerhof (GGA-PBE) was adopted for the exchange-correlation functional^46^. Moreover, the electron wave functions were expanded with a plane wave cutoff of 400 eV. The (10 × 10 × 1) supercell containing 200 carbon atoms was constructed using periodic boundary conditions (PBCs), and the vacuum layers were set to be larger than 20 Å to avoid periodic interactions. Reciprocal space integration was performed by the Monkhorst-Pack special k-point scheme with 2 × 2 × 1 grid meshes for the structure relaxation for the Pt adsorption mesoporous carbon (MC). Atomic relaxation was performed until the total energy variation was less than 10^−6^ eV and all forces on each atom were less than 0.01 eV Å^−1^. The DFT-D3 approach was used to evaluate the effect of the van der Waals (vdW) interaction^47^. Bader charge analysis^48^ was performed to quantitatively estimate the amount of charge transfer between the adsorbed Pt (or H) and the mesoporous carbon. To investigate whether Pt will cluster on the anatase (101) surface, the adsorption energies of Pt on a (1 × 4) repeated surface supercell (10.35 Å × 15.21 Å) were calculated. Oxygen vacancies (O_v_) generally exist in anatase TiO_2_, and all the models used in the calculations contained an O_v_ defect.

The first-principles molecular dynamics (FPMD) calculations were performed using the CP2K/QUICKSTEP package^49^. The wave functions of the valence electrons were expanded in terms of Gaussian functions with molecularly optimized double-ζ polarized basis sets (m-DZVP), ensuring a small basis set superposition error^50^, and core electrons were described with norm-conserving Goedecker, Teter, and Hutter (GTH) pseudopotentials^51^. The FPMD was carried out with the NVT ensemble at the target temperature of 300 K or 1000 K using the Nose−Hoover thermostat. The time step in all calculations was 1 fs, and each sample was equilibrated at the target temperature for 50 ps, which is considered long enough for nucleation or reactions on MC or metal oxides.

### Data availability

All data are available from the authors upon reasonable request.

## Electronic supplementary material

Supplementary Information
