# Supplementary material for: Iced photochemical reduction to synthesize atomically dispersed metals by suppressing nanocrystal growth
Source: Nat Commun. 2017 Nov 14;8:1490. doi: 10.1038/s41467-017-01521-4 (PMC5684195; doi:10.1038/s41467-017-01521-4)
Supplement: Supplementary file 1 — Supplementary Information [file 41467_2017_1521_MOESM1_ESM.pdf]

Supplementary Methods

1. Materials.

Chloroplatinic acid (H_2PtCl_6), silver nitrate ($AgNO_3$), and chloroauric acid ($HAuCl_4$) were purchased from Sinopharm Chemical Reagent Co. Ltd. Graphene powder Physical methods was purchased from Nanjing XFNANO Materials Tech Co. Ltd. Commercial Pt/C (20 wt%) was purchased from Johnson Matthey. Ultrathin carbon film on holey carbon (400 mesh, Cu) was purchased from Ted Pella Inc. All chemical materials were used without further pretreatment.

2. Preparation of the Pt single atoms solution.

A 0.3 mg ml^{-1} H_2PtCl_6 solution was prepared using chloroplatinic acid and deionized water. And a 1.5 ml H_2PtCl_6 solution was frozen quickly and entirely using liquid nitrogen. Then the ice irradiated by UV lamp for 1 hour, meanwhile, the environment was kept in -25 ℃ in a lyophilizer (LGJ-10N), avoiding the melt of ice. The powder density of the UV light near ice was measured (0.89 mW cm^{-2}) using a radiometer (PL-MW 2000). After UV irradiation, the H_2PtCl_6 ice was melted at room temperature. These treatments were all kept in a dark environment except of the UV irradiation. Therefore, the Pt single atoms solution was prepared. The concentration of Pt precursor in our system (0.73 mM) was comparable to that extensively used for the solution-based syntheses of Pt nanoparticles (Supplementary Table 7). Meanwhile, we have carefully considered the distribution of light in the solid ice in our iced-photochemical process and discovered an appropriate solution by trial and error. It was found that the UV light can effectively transmit the frozen H_2PtCl_6 solution when the thickness of ice layer was thin enough (Supplementary Fig. 29). To achieve this goal, we used a flat-bottomed container

with a large capacity relative to the volume of solution. For example, the thickness of frozen solution was just ~1.3 mm when a 3 mL of H_2PtCl_6 solution was put into the container (55 mm in diameter).

3. Preparation of the Pt_1/MWCNT and Pt_1/G.

A 10 ml Pt single atoms solution and a 10 ml mg ml^{-1} multi-walled nanotubes (or graphene) solution were mixed, filtered, cleaned and dried at room temperature. Then, the Ag_1/MC powders were collected.

4. Preparation of Pt_1/TiO_2 and Pt_1/ZnO.

A 10 mL Pt single atoms solution and 1 mg TiO_2 (or ZnO) were mixed together. The mixed solution was filtered, cleaned and dried at room temperature.

5. Preparation of Ag single atoms solution and Ag_1/MC.

A 0.3 mg ml^{-1} $AgNO_3$ solution was frozen quickly and entirely using liquid nitrogen, followed by UV irradiation (0.89 mW cm^{-2}) for one hour at -25 ℃ in a lyophilizer. Then the Ag single atoms solution was obtained after the ice melting process at room temperature in a dark place. A 10 ml mesoporous carbon solution (5 mg ml^{-1}) and a 25 ml the obtained Ag single atoms solution were mixed. Then, the Ag_1/MC samples were cleaned and dried at room temperature.

6. Preparation of Au single atoms solution and Au_1/MC.

A 0.1 mg ml^{-1} $HAuCl_4$ solution was frozen quickly and entirely using liquid nitrogen. The subsequent processes of Au_1/MC were similar to these of Ag_1/MC.

7. X-ray diffraction (XRD) characterizations.

XRD (D/max 2500 V) was performed with a Cu target and 8 °min^{-1} scanning speed. And the operation voltage and current were 40 kV and 200mA, respectively.

8. TEM characterizations.

TEM images were measured by a JEOL-2010 microscope. The samples were prepared by dropping 15 ul of the Pt single-atom solution onto 400 mesh ultrathin carbon film on a carbon support film with holes. The ultrathin carbon film was dried at room temperature in dark place and cleaned with deionized-water to remove any remaining unreactive H_2PtCl_6. Then, the cleaned ultrathin carbon film was dried in a dark environment.

9. STEM characterizations.

Aberration-corrected high-angle annular dark field-STEM images were acquired using a JEM-ARM200F transmission electron microscopy operated at 200 kV. Electron energy loss spectroscopy was performed to ensure the elements of Pt single atoms and Pt nanoparticles. The energy at 2122 eV corresponded to Pt element for Pt single atoms and Pt nanoparticles.

10. STM characterizations.

The Asylum Research Cypher was used to perform the STM tests under ambient conditions (temperature 20 to 25 ℃, relative humidity ~30 %) using a platinum-iridium (Pt-Ir) alloy tip under the constant-current mode.

11. XPS characterization.

X-ray photoelectron spectroscopy measurements were performed using an X-ray photoelectron spectrometer (Escalab 250Xi) equipped with an Al Kα radiation source (1487.6 eV) and hemispherical analyzer with a pass energy of 30.0 eV and an energy step size of 0.05 eV. The collected data were corrected for charging effect-induced peak shifts using the binding energy (BE) of C 1s peak of substrate (284.8 eV). Spectral

deconvolution was performed by Shirley background subtraction using a Voigt function convoluting the Gaussian and Lorentzian functions.

12. ICP-MS characterization.

The Pt loading contents of Pt single atoms materials were measured and confirmed using ICP-MS measurements (ELAN DRC-e). Confirmed by ICP-MS test, the Pt loading of Pt_1/MC was 2.6%, which can be regarded as a relatively high level compared with other single Pt atom catalysts. As shown in Table S8, Pt metal remained atomically dispersed with a low Pt loading (< 2 wt.%) while Pt clusters or nanoparticles from the aggregation of single atoms emerged with the increase of Pt loading.

Supplementary Notes

Supplementary Note 1. The stable adsorption configuration of H-Pt-OH on MC.

The stability and configuration of H-Pt-OH adsorption on the common defects or edges in MC were carefully examined based on first-principles calculations. The energy difference between the H-Pt-OH adsorption and the H_2O-Pt adsorption on MC, ΔE_r, was calculated. The total energy of the H-Pt-OH adsorption was set as a reference energy. In this scheme, a relatively large negative value, ΔE_r, indicates the formation of H_2O-Pt single atoms adsorption on MC is more stable than H-Pt-OH adsorption on MC. The results show that ΔE_r for SV and DV are -0.345 and -1.105, respectively (Supplementary Fig. 14). The results clearly indicate that the H-Pt-OH could be easily transformed into Pt single atom by forming water on MC.

Supplementary Note 2. Adsorption of Pt single atom on mesoporous carbon and graphene.

The DFT calculations were carried out using a graphene (10 × 10 × 1) supercell containing 200 C atoms to identify the adsorption strengths of single Pt atom on the common defects, such as SV, DV, and edges. Considering the existence of all kinds of defects of mesoporous carbon in experiment, the Pt single atoms adsorbed on the defects and edges were carefully considered. All inequivalent Pt-adsorption configurations at these defects or edges have been investigated, and the most stable configurations are shown in Supplementary Fig. 13. The adsorption energies (E_a) for each configuration were calculated using the following equation:

$$E_a = \frac{1}{n}(E_M + nE_{Pt} - E_{M+nPt}) \quad (1)$$

Where E_{pt} is the energy of an isolated Pt atom, E_M is the total energy of the defects/edges systems, and E_{M+nPt} is the total energy of the Pt adsorption on substrates with defects or edges, respectively. A positive E_a indicates the stable Pt adsorption. As shown in Supplementary Table 4, all the adsorption energies are positive for the Pt adsorption on perfect carbon, defects and edge sites, indicating the stability of the Pt single atoms on these systems. As for the Pt single atoms adsorbed on perfect graphene, almost no charge transfer occurs between Pt and graphene. Different from the perfect graphene, the large charge transfer appears between Pt and C in the SV/DV and the edge, which confirms the relatively large Pt adsorption energy at these defect/edge sites.

Supplementary Note 3. Stability of Pt single atoms on mesoporous carbon and graphene.

To further examine the distribution of the Pt atoms on the mesoporous carbon, the energy differences between the isolated Pt atom and the Pt cluster, ΔE_c, at both defects and edge sites were calculated. Here the total energy of the isolated configuration was used as a reference energy. In this scheme, a relatively large positive value of ΔE_c indicates that Pt

atoms energetically prefer to adsorbed as the single-atom instead of clustering. For comparison of substrates, various possible Pt cluster configurations on pristine graphene were also examined.

Supplementary Note 4. Oxidation state of Pt on the MC substrate.

We further characterized the oxidation state of Pt on the MC substrate before and after HER catalytic step using first-principle calculations. As shown in Supplementary Fig. 30 and Supplementary Table 9, when the Pt single atom adsorbed on the typical SV of MC, two d orbital, d_{yz} and d_{xz} became partially occupied, and the corresponding occupation numbers were 1.365 *e* and 1.367 *e*. As a result, the original Pt ($6s^15d^9$) electron configuration changed to Pt ($6s^15d^8$) with the +1 oxidation state of Pt. After the Volmer step ($H^+ + e^- \rightarrow H^*$) (Supplementary Fig. 30b), the adsorption of two hydrogen protons made another orbital (dz^2) partially unoccupied, whose occupation number dropped from 1.864 *e* to 1.450 *e*. This eventually electron structures lead to the change of Pt valence-state from +1 to +2 after hydrogen adsorption. Similarly, the change of Pt oxidation state in the Pt adsorption DV in MC was also analyzed. The adsorption of Pt on DV lead to the generation of two half-occupied *d* orbitals with their occupation numbers of 1.487 *e* and 1.426 *e*. In this case, the oxidation state of Pt was determined to be +1. However, upon the adsorption of two hydrogen protons, one more d orbital became half-occupation (1.555 *e* by the partial charge analysis), indicating that Pt became +2 valence. All our calculation results agreed well with the XPS results, which suggested the $Pt^{\delta+}$ [1, 2] in Pt_1/MC. Therefore, it is greatly interesting but remains a quite challenge to determine the oxidation state of H-Pt-OH by either theory or experiment.

Supplementary Note 5. DFT calculation of HER catalytic performances.

The overall HER pathway ($H^+ + e^- \rightarrow 1/2H_2$) can be divided into two separate pathways comprising either the Volmer-Heyrovsky or the Volmer-Tafel mechanism [3, 4]. Both these two mechanisms can be summarized by a three-state diagram, composed by an initial state $H^+ + e^-$, an intermediate adsorbed H^*, and a final product $1/2H_2$. The Gibbs free energy of the intermediate state, $|\Delta G_{H*}|$, is regarded as a major indicator of the HER activity for various catalysts, which can be calculated by the following equation:

$$\Delta G_H = \Delta E_H + \Delta E_{ZPE} - \Delta S_H T \tag{2}$$

where ΔS_H is the difference in entropy. ΔE_{ZPE} is the difference in zero-point energy between the adsorbed and the gas phase. $\Delta E_{ZPE} - \Delta S_H T$ is about 0.24 eV [5]. ΔE_H is the hydrogen chemisorption energy defined as:

$$\Delta E_H = \frac{1}{n}(E_{MC+nH} - E_{MC} - \frac{n}{2} E_{H_2}) \tag{3}$$

where n is the number of H atoms in the calculation. The most desirable value for $|\Delta G_{H*}|$ should be zero [6]. A (2 × 2) Pt (111) were used to calculate the catalytic activity of the typical Pt (111) catalyst. The calculated free energy diagram for hydrogen adsorption on Pt (111) was consistent with the previous report [7] (Supplementary Fig. 27).

Supplementary Figures

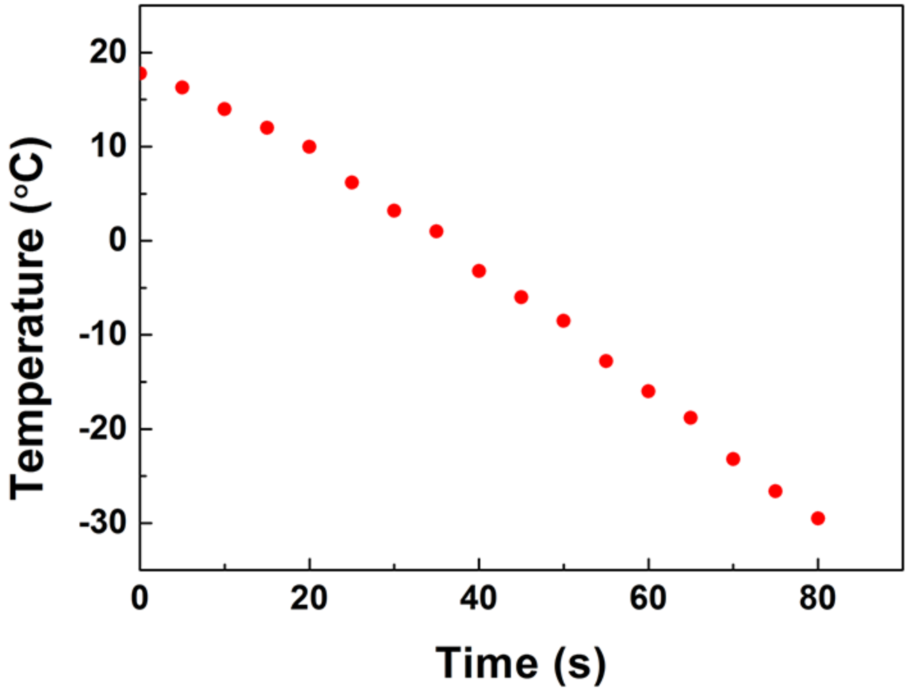

Supplementary Figure 1. The temperature changes versus time while freezing the H_2PtCl_6 solution in liquid N_2. The solution was frozen by dipping the container in liquid nitrogen, while the temperature of the solution was recorded using the system's test digital thermometer. The temperature decrease rate is ~ 40 ℃ min^{-1}.

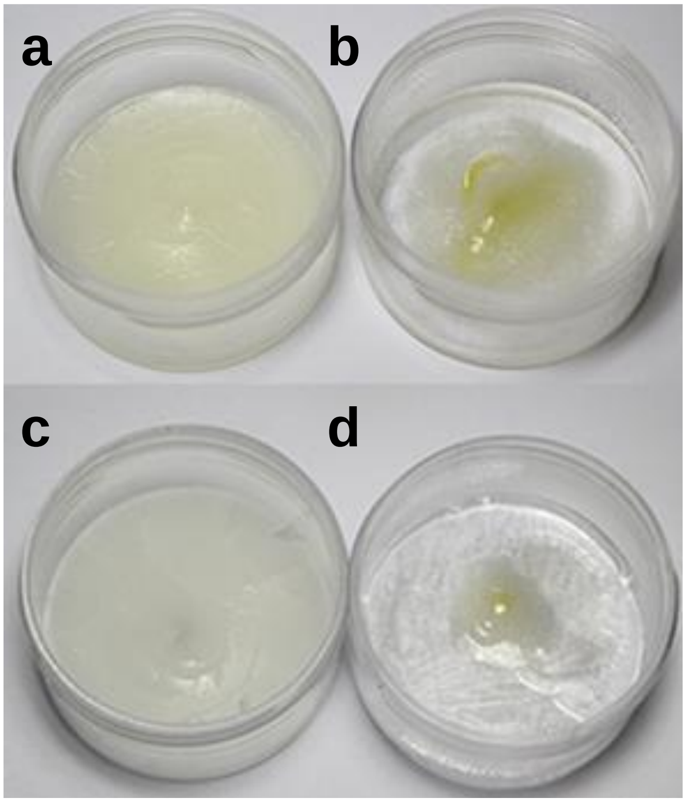

Supplementary Figure 2. Photograph of frozen H_2PtCl_6 solution. (a) A 1 mg ml^{-1} H_2PtCl_6 solution was frozen using liquid nitrogen, (b) A 1 mg ml^{-1} H_2PtCl_6 solution was frozen using a refrigerator, (c) A 0.3 mg ml^{-1} H_2PtCl_6 solution was frozen using liquid nitrogen, and (d) A 1 mg ml^{-1} H_2PtCl_6 solution was frozen using a refrigerator. In order to illustrate obviously, much more solution than normal experiment was used here.

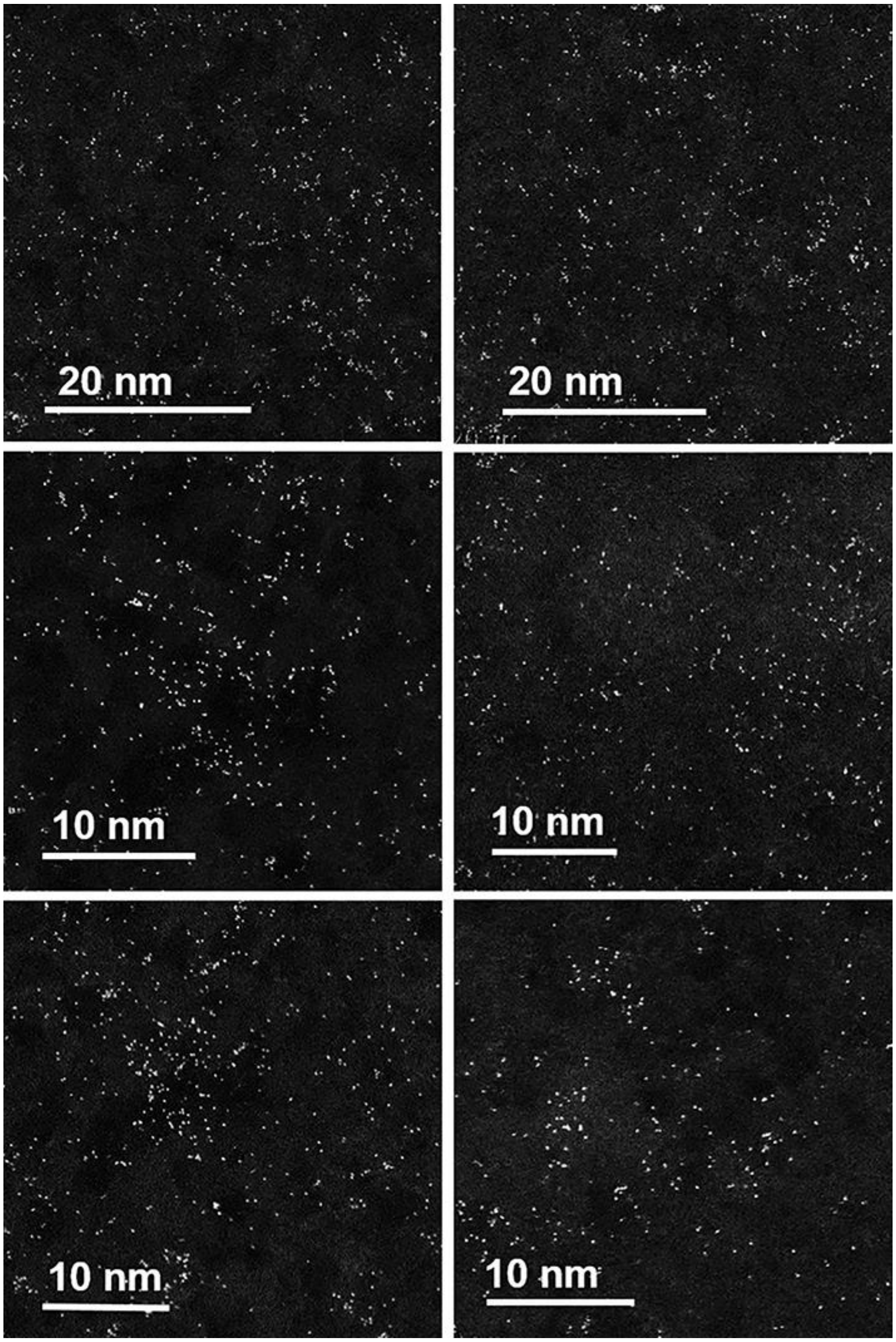

Supplementary Figure 3. STEM images of Pt_1/C. Densely and homogeneously dispersed Pt on ultrathin carbon film can be clearly observed with absence of large Pt clusters.

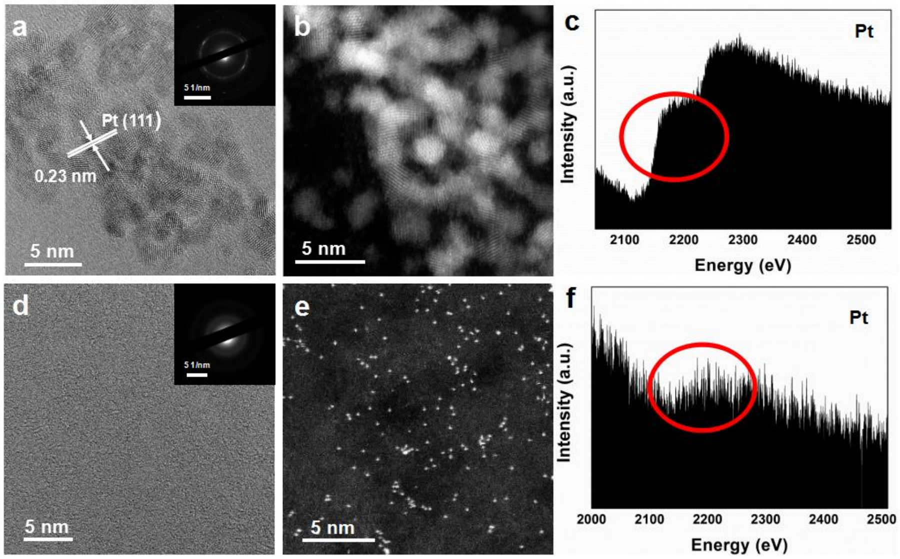

Supplementary Figure 4. TEM, STEM images and EELS of Pt nanoparticles and Pt single atoms. (a) TEM image of Pt nanoparticles, interplanar spacing with 0.23 nm corresponding to Pt (111). (b) STEM images of Pt nanoparticles, particles size with 2-3 nm. (c) EELS of Pt nanoparticles, corresponding to Pt element. (d) TEM image of Pt single atoms, without Pt nanoparticles. (e) STEM image of Pt single atoms, with atomically dispersed bright dots. (f) EELS of Pt single atoms, corresponding to Pt element.

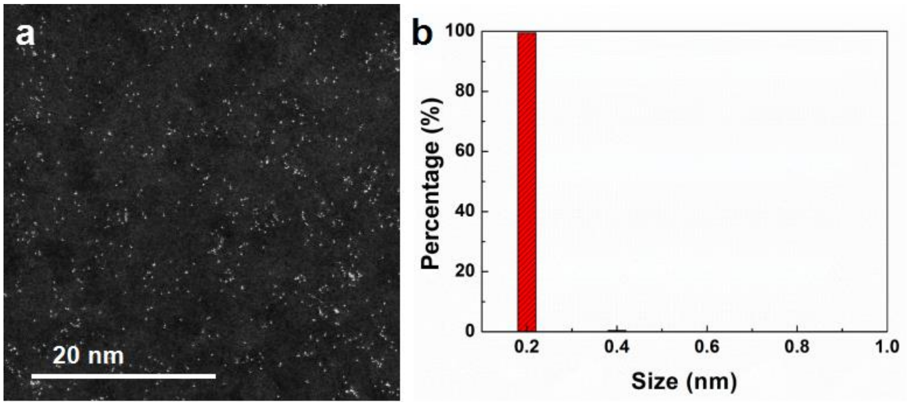

Supplementary Figure 5. Size distribution of Pt single atoms. (a) STEM image of Pt single atoms loaded on carbon. (b) The size distribution of Pt single atoms loaded on carbon shows that the percentage of Pt single atoms in STEM image is more than 99%.

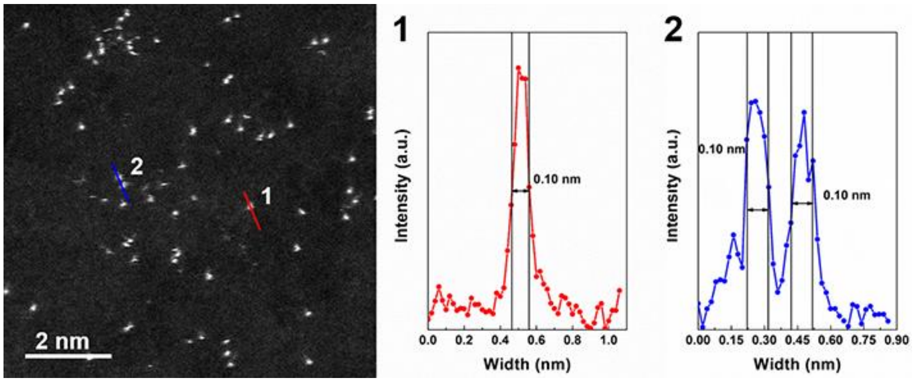

Supplementary Figure 6. Size of Pt single atoms. The size of Pt single atoms is about 0.1 nm.

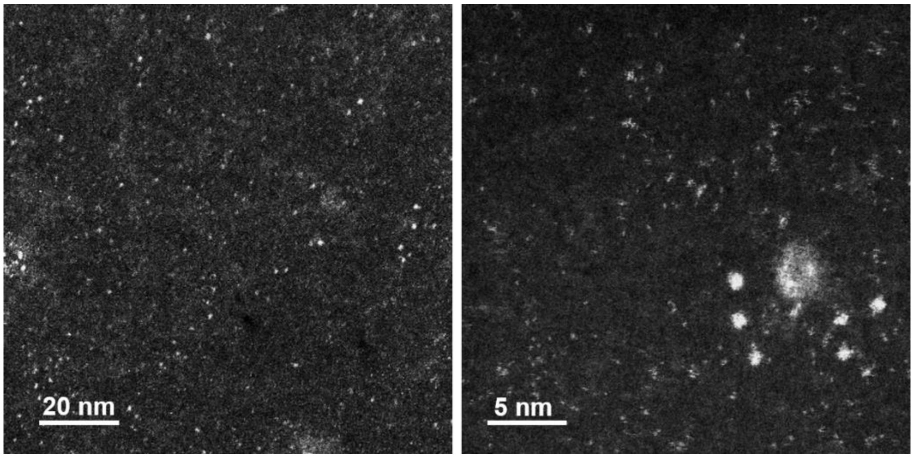

Supplementary Figure 7. STEM images of Pt single atoms and clusters on ultrathin carbon film using 3 mg ml^{-1} H_2PtCl_6. The Pt atoms became partially aggregated at this concentration.

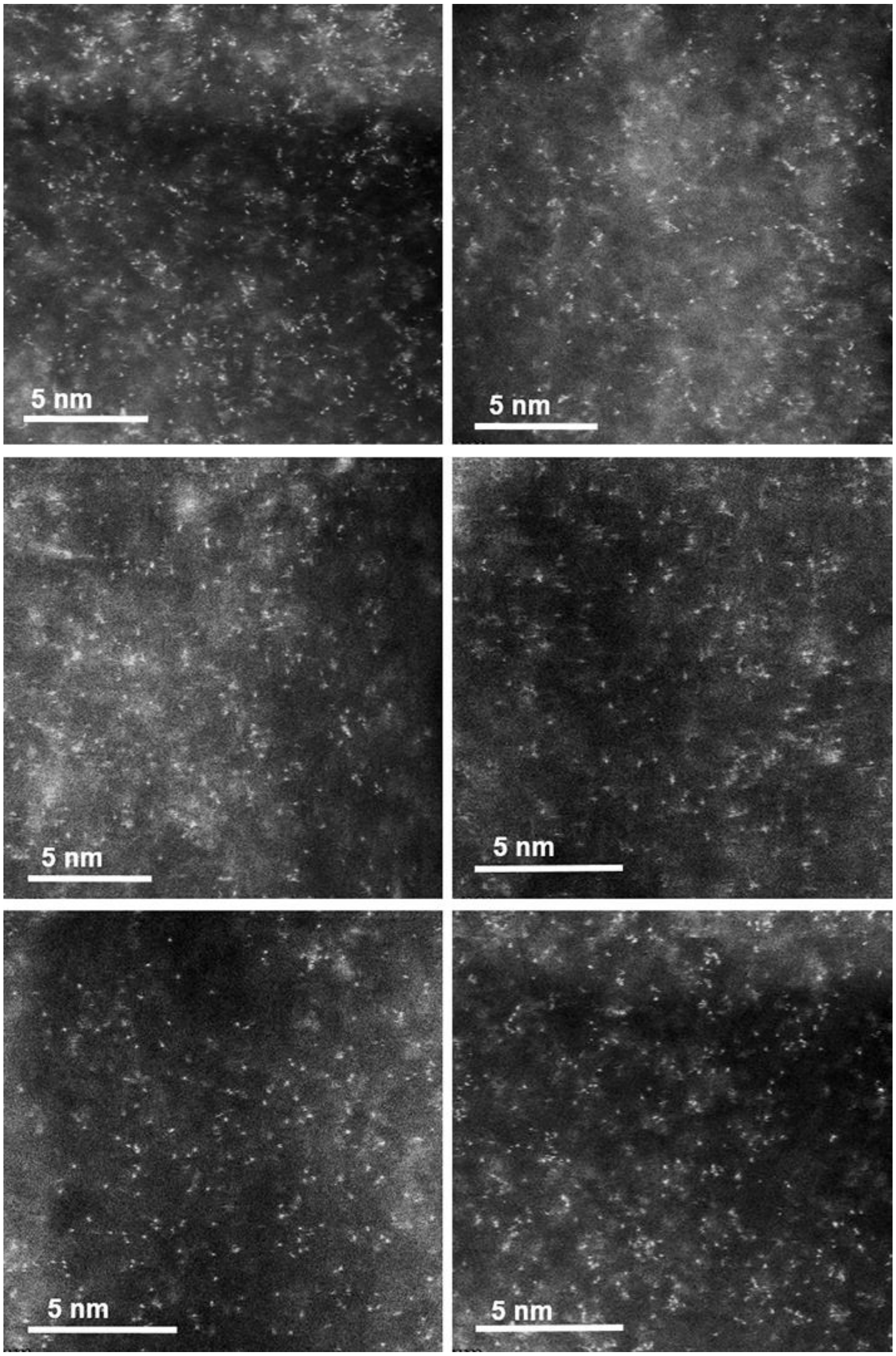

Supplementary Figure 8. STEM images of Pt_1/MC on difference areas and different scales. Densely and homogeneously dispersed Pt on mesoporous carbon can be clearly observed with absence of large Pt clusters.

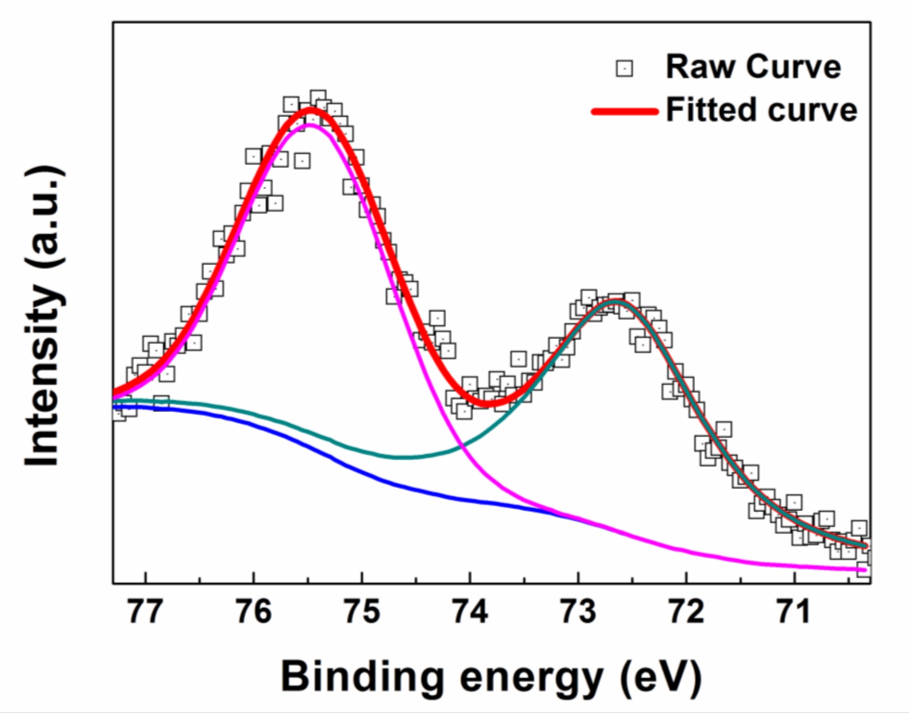

Supplementary Figure 9. XPS characterization of Pt_1/MC. The binding energy of Pt $4f_{7/2}$ and $4f_{5/2}$ for Pt_1/MC is corresponding to $Pt^{\delta+}$, indicating the positive charge on Pt for Pt_1/MC.

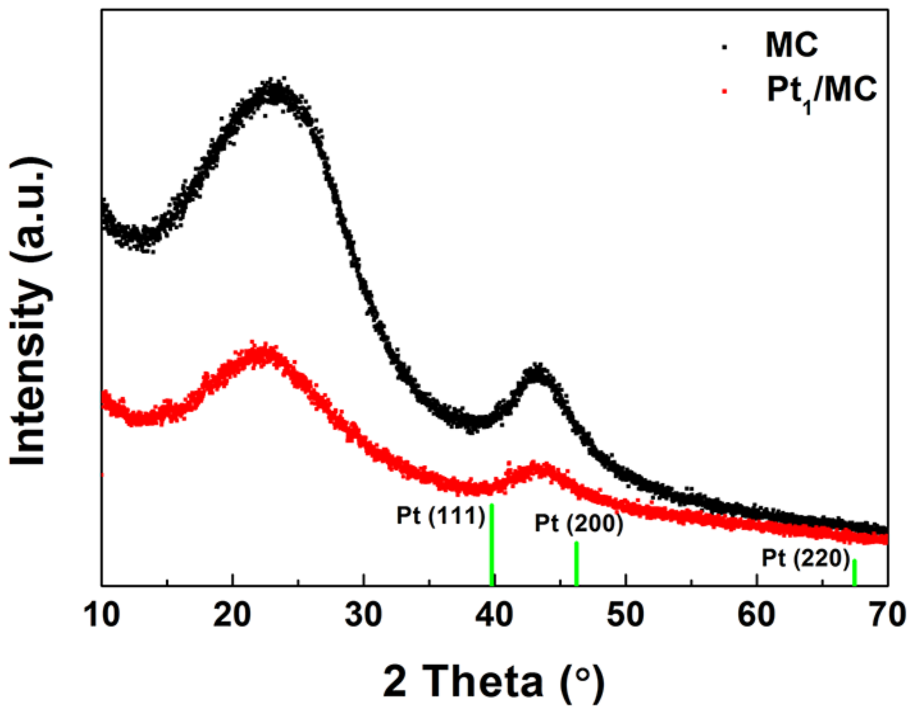

Supplementary Figure 10. XRD results of MC and Pt_1/MC. No peak of Pt lattice has been detected in the sample.

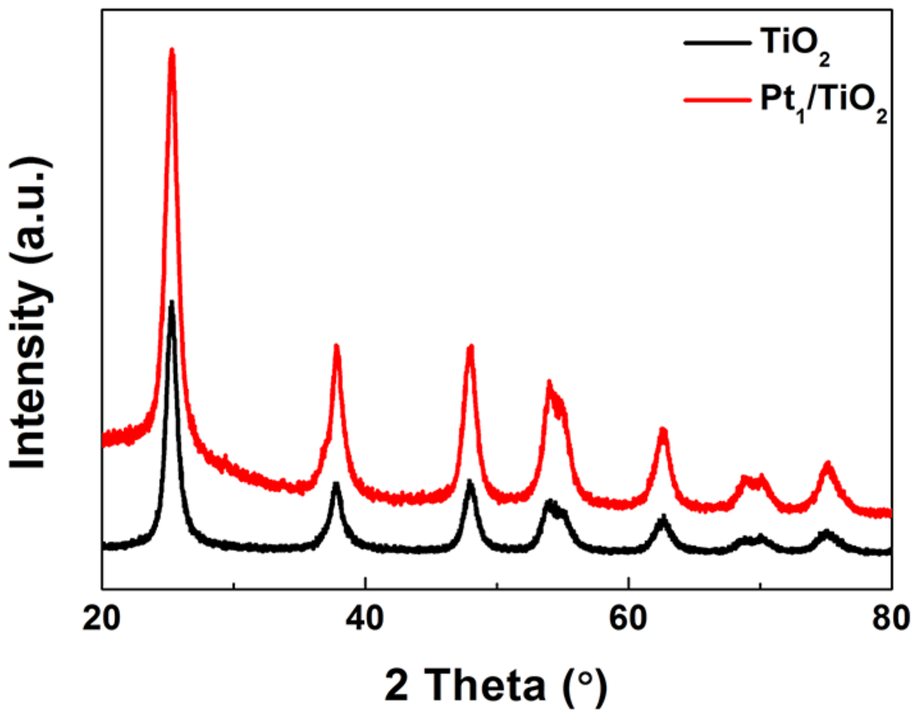

Supplementary Figure 11. XRD pattern of TiO_2 and Pt_1/TiO_2. No diffraction peaks for Pt nanoparticles have been detected.

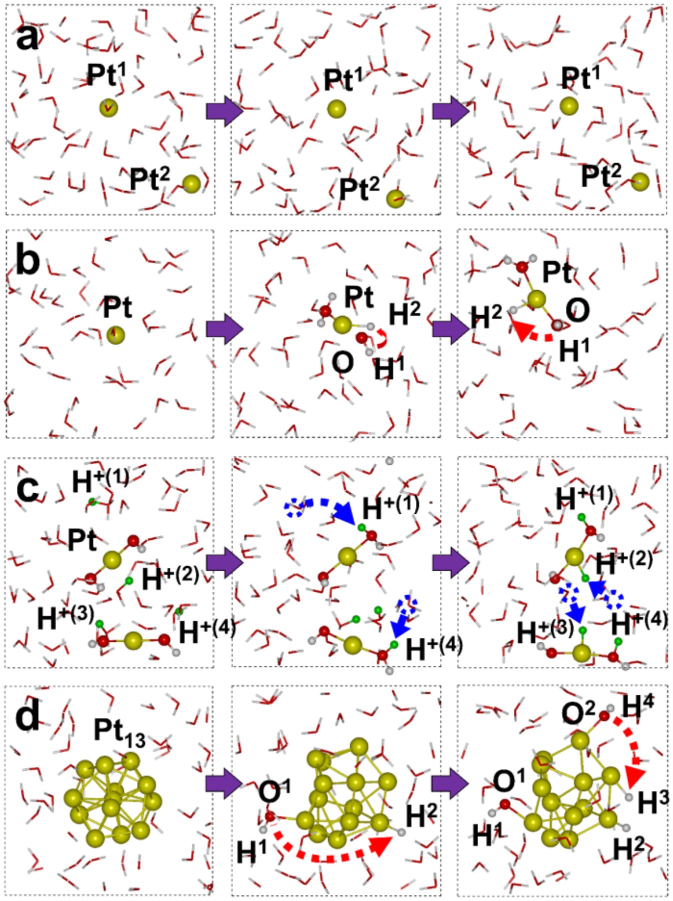

Supplementary Figure 12. The stabilities of Pt single atom, $Pt(OH)_2$ and Pt cluster in aqueous solution. The Pt single atom in water environment under (a) the room temperature and (b) 1000K. The stability of (c) $Pt(OH)_2$ compound and (d) Pt_{13} cluster in water environment under the room temperature. The initial, intermediate and final configurations of each process selected from the FPMD simulations were shown at the left, middle and right of the diagram. In order to show how the H-Pt-OH evolves, the water molecules react with the Pt atoms are highlighted. The yellow, red, white and green balls indicate Pt, O, H atoms. Here Pt^n (n = 1-2) and H^n (n = 1-4) represent the different Pt and hydrogen atoms, respectively. The red and blues dot lines represent the direction of proton diffusion during H_2O dissociation and proton transfer, respectively.

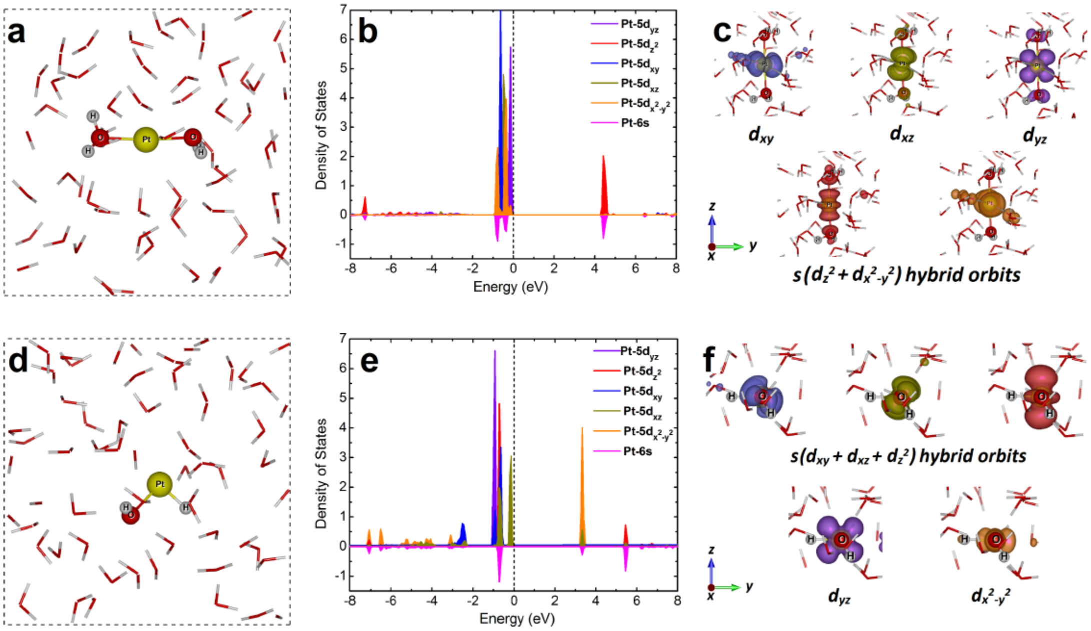

Supplementary Figure 13. (a), (d) The illustrated structure, (b), (e) partial density of states (PDOS) and (c), (f) partial charge of the Pt single atom in the form of Pt-H_2O and H-Pt-OH in water environment, respectively. Here, atomic configurations of (a) and (d) were randomly selected from the first-principles molecular dynamics simulations, and (a) was taken from the beginning stage of the simulation, and the final stage of the simulation. The yellow, red and white balls indicate Pt, O, H, respectively. The positive y-axis shows the PDOS of Pt-d orbitals in Pt-H_2O and H-Pt-OH systems, and the negative y-axis exhibits the PDOS of Pt-s orbitals. The Fermi level is shifted to zero. The suborbitals of the Pt-d near the Fermi level in Pt-H_2O and H-Pt-OH are shown in (c) and (f), respectively.

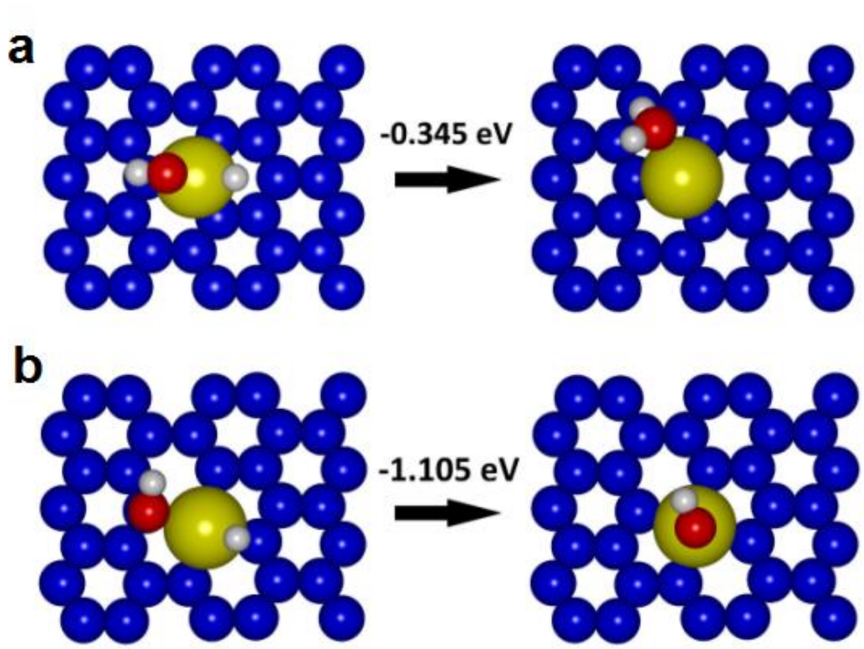

Supplementary Figure 14. The structure of H-Pt-OH on mesoporous carbon. The detachment process of H and OH from the Pt single atoms which are adsorbed on the typical (a) single vacancy and (b) double vacancy, respectively. These results indicate that Pt+H_2O is more stable than H+Pt+OH in both cases.

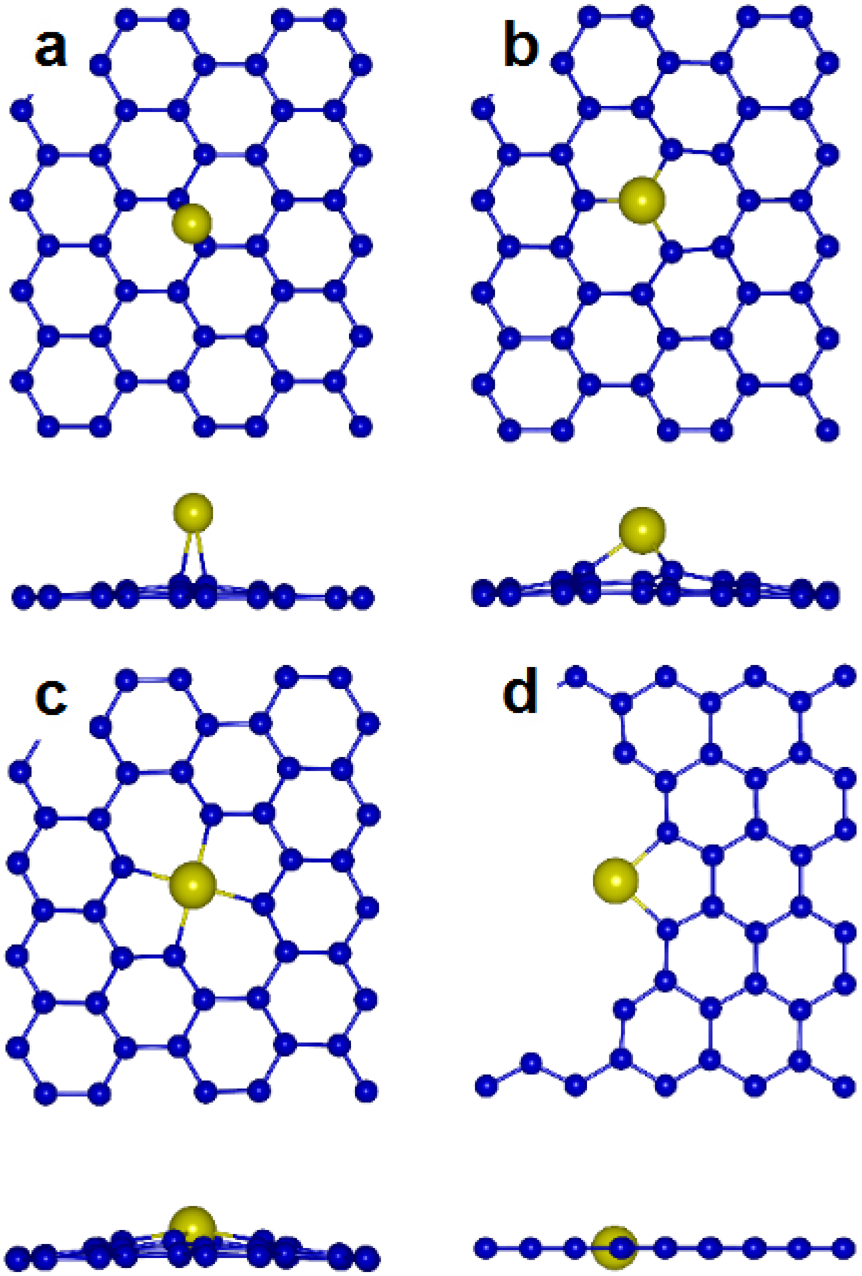

Supplementary Figure 15. Structures of Pt single atom adsorbed on the perfect graphene and the defects/edges sites of graphene. (a) Perfect graphene (PG). (b) Single vacancy. (c) Double vacancy. (d) Edge sites.

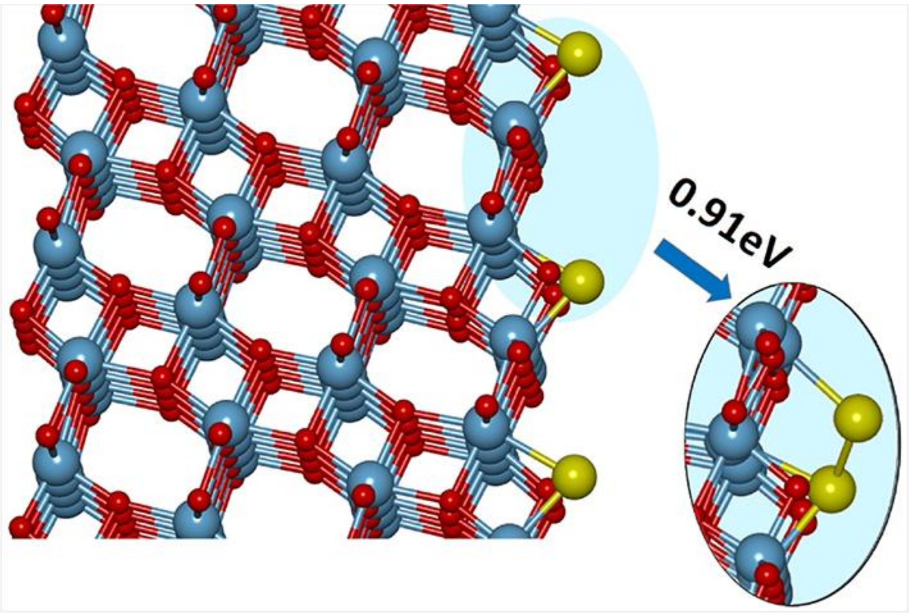

Supplementary Figure 16. Structure and size distribution for Pt single atoms and Pt clusters on anatase TiO_2 (101) surface.

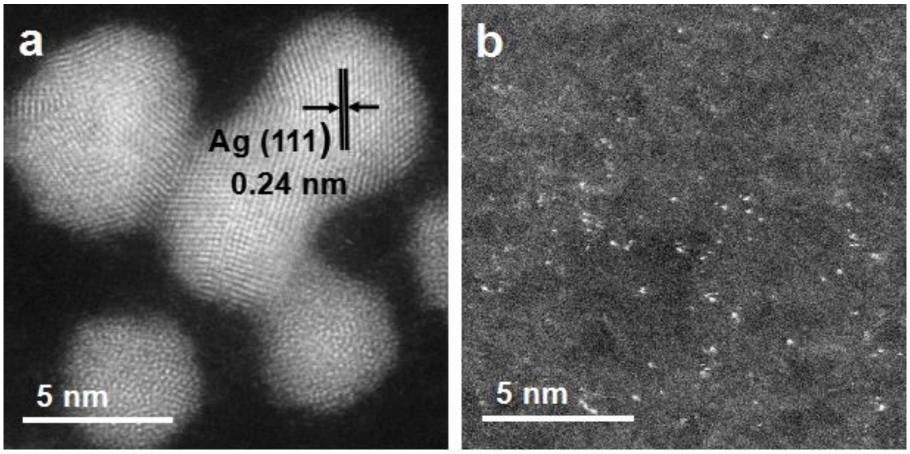

Supplementary Figure 17. The comparison of STEM images of Ag nanoparticles and Ag single atoms. (a) Ag nanoparticles fabricated with conventional $AgNO_3$ photo reduction. The interplanar spacing of 0.24 nm corresponded to Ag (111). (b) Atomically dispersed Ag produced with the iced-photochemical method.

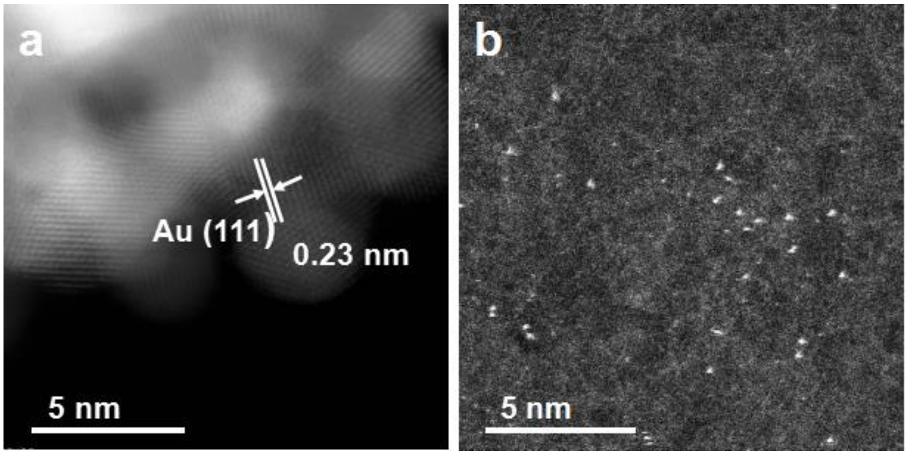

Supplementary Figure 18. The comparison of STEM images of Au nanoparticles and Au single atoms. (a) Au nanoparticles fabricated with conventional photochemical reduction. The interplanar spacing of 0.23 nm corresponded to Au (111). (b) Atomically dispersed Au produced with the iced-photochemical method.

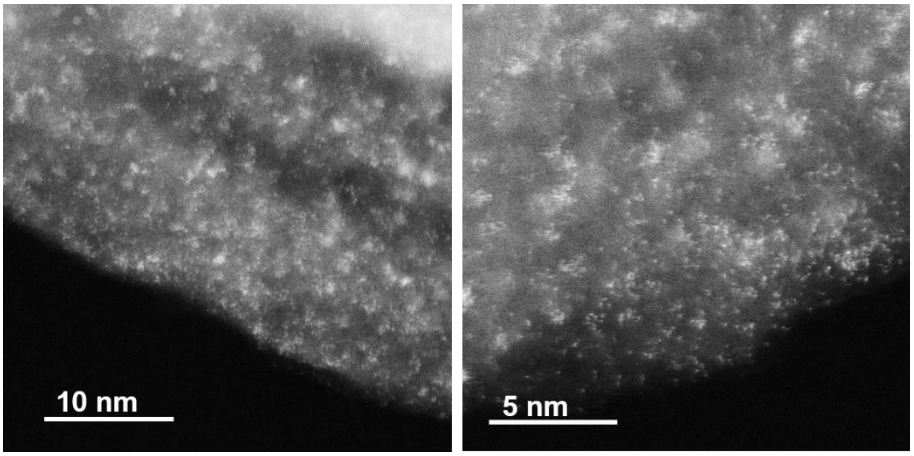

Supplementary Figure 19. STEM images of Pt_1/MC with the Pt loading of 2.6 wt.%.

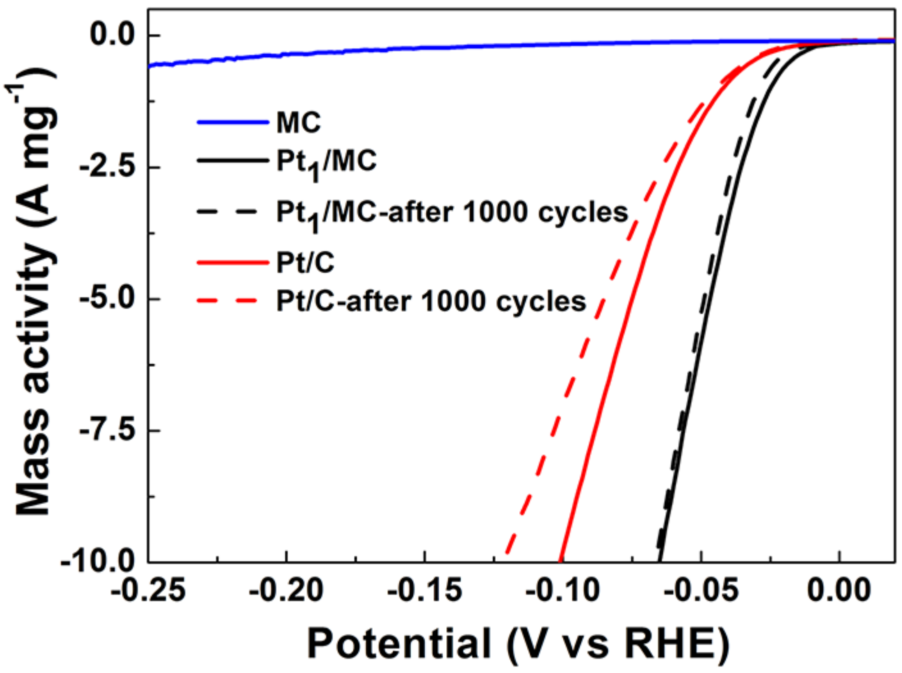

Supplementary Figure 20. Mass activity of Pt_1/MC, Pt/C and MC before and after 1000 CV cycles. The mass activity of Pt_1/MC is superior to traditional Pt/C electrode.

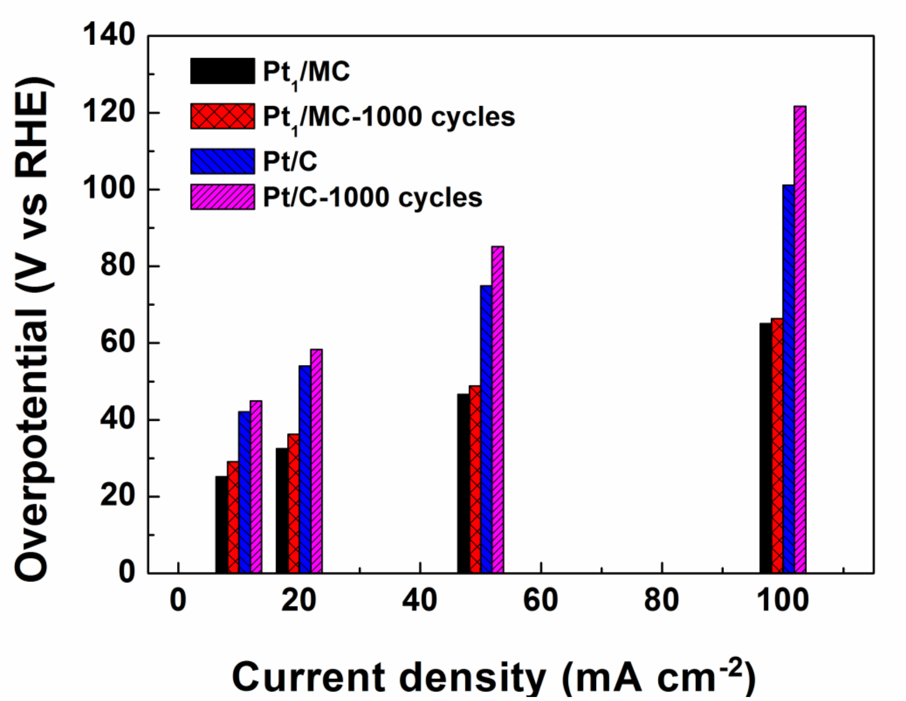

Supplementary Figure 21. Overpotential of Pt_1/MC and Pt/C electrocatalysts for HER at certain current density. The overpotential of Pt_1/MC is lower than that of Pt/C at the same current density, especially after 1000 CV cycles. It indicates the HER performance of Pt_1/MC is superior to that of Pt/C.

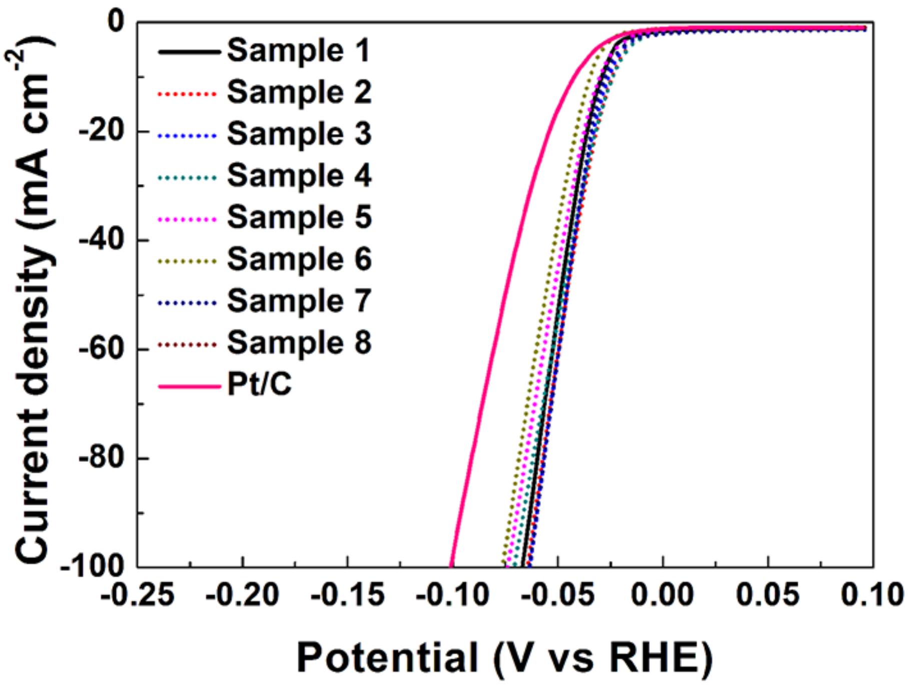

Supplementary Figure 22. HER activity of different Pt_1/MC samples with same Pt mass loading. The performance of the Pt_1/MC electrocatalyst is reproducible.

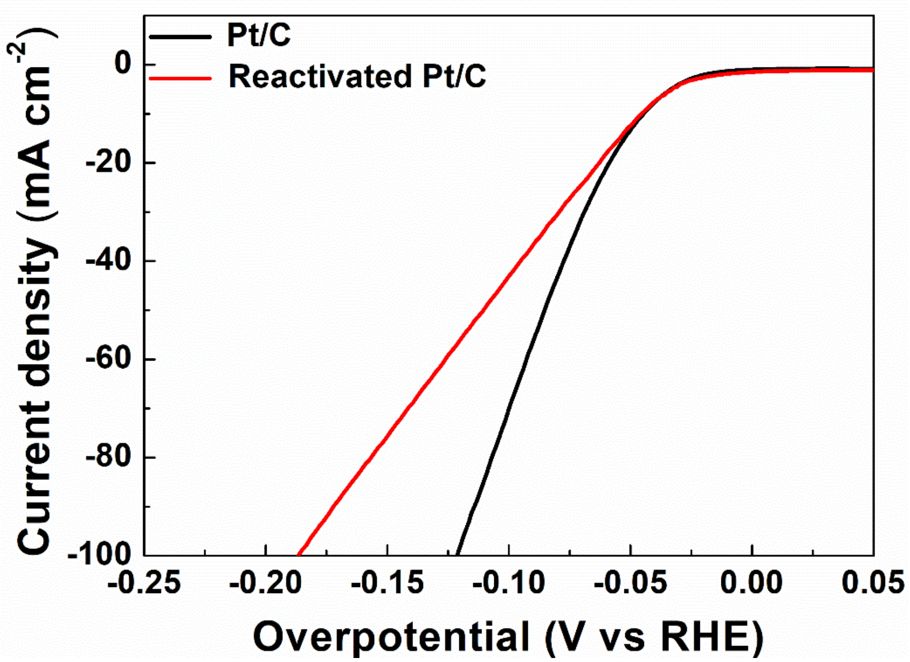

Supplementary Figure 23. The linear sweep voltammetry (LSV) curves for initial Pt/C and Pt/C reactivated by CV cycles after long-term stability measurement.

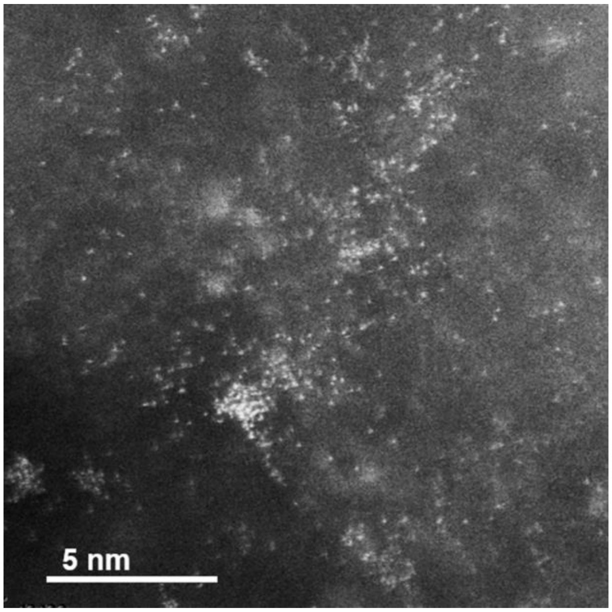

Supplementary Figure 24. STEM images of Pt_1/MC after 1000 cycles CV stability.

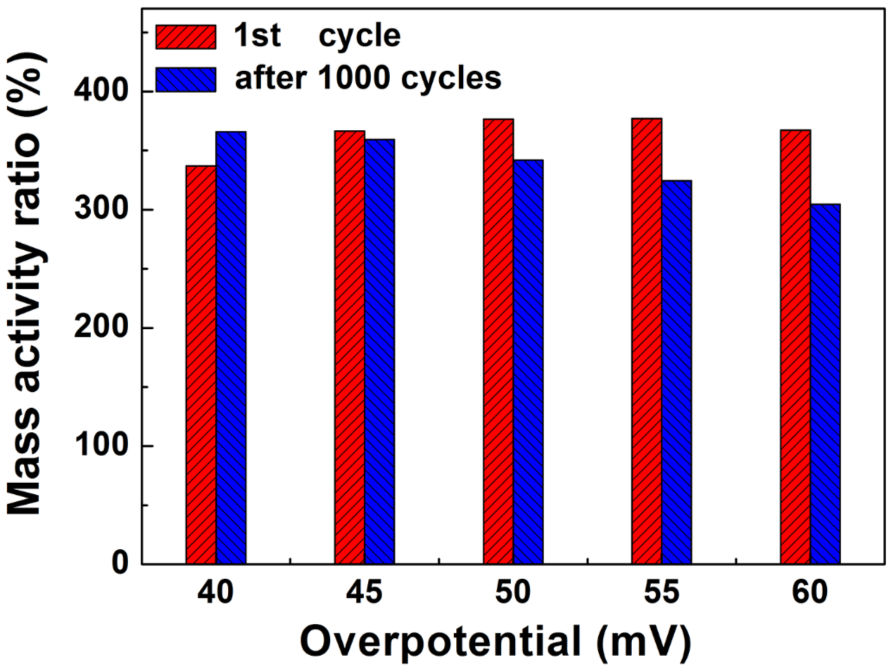

Supplementary Figure 25. Mass activity ratio between Pt_1/MC and Pt/C (collected before and after 1000 CV cycles).

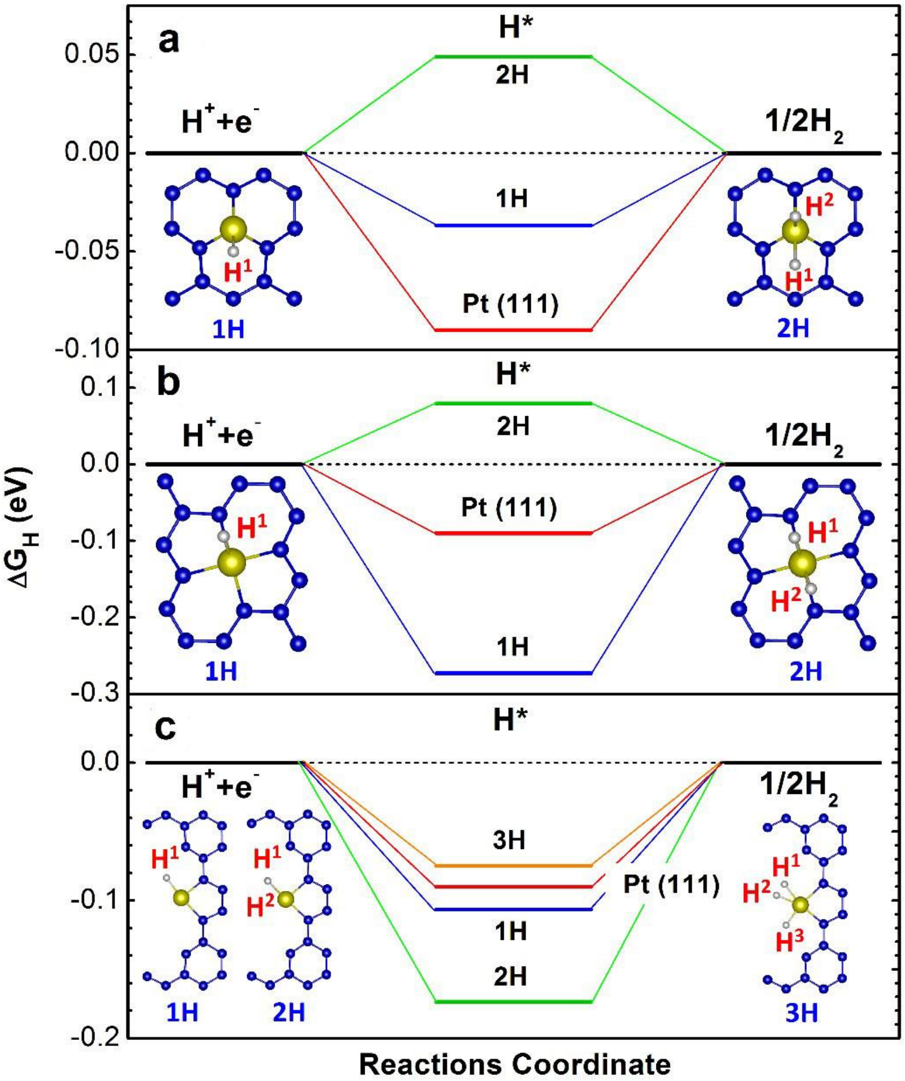

Supplementary Figure 26. The DFT optimized structures and the free energies (ΔG_H) for different H concentrations on (a) single-vacancy, (b) double vacancy and (c) hole edge MC systems, respectively. The yellow, red and white balls indicate Pt, O, H, respectively. Only half of the hole edge MC model is shown to save the space, the detailed model of the hole can be seen in Fig. 3.

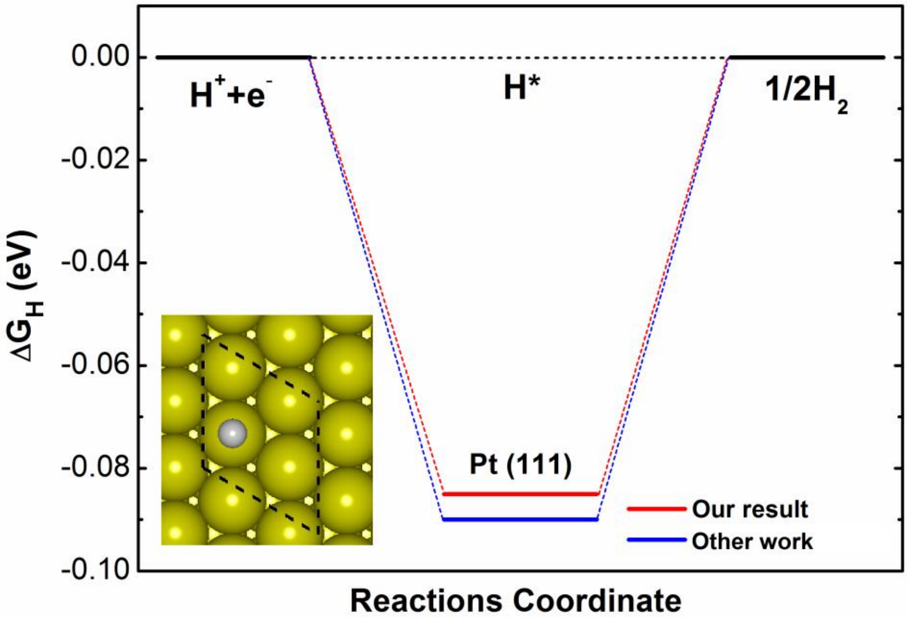

Supplementary Figure 27. Free energy diagram for hydrogen evolution on Pt (111) from our calculation and by other work[7].

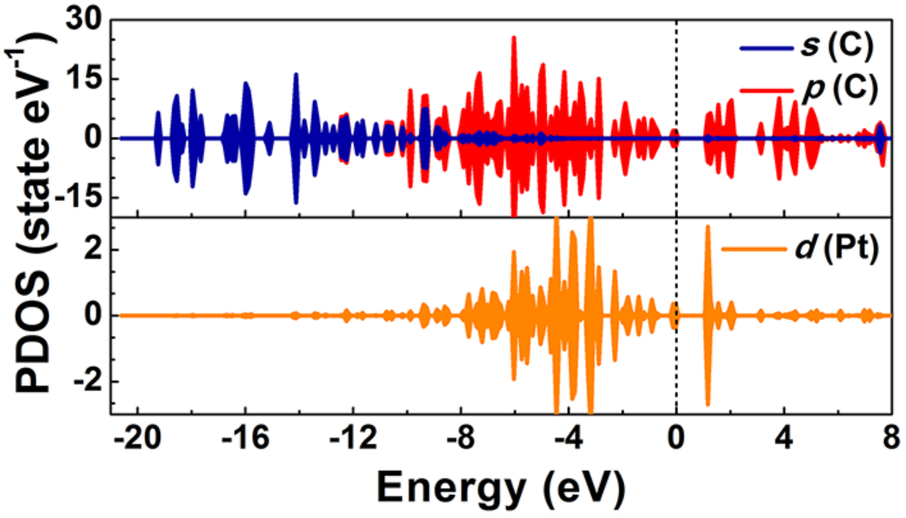

Supplementary Figure 28. The partial density of states (PDOS) of the Pt adsorbed on single vacancy system before H adsorption. The Fermi level is shifted to zero. The panel shows the PDOS of carbon atoms.

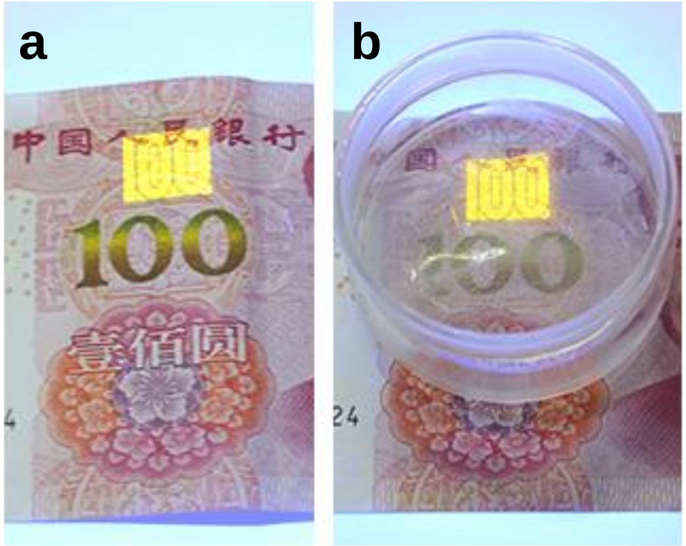

Supplementary Figure 29. Photographs of photosensitive material without (a) and with (b) ice during UV irradiation.

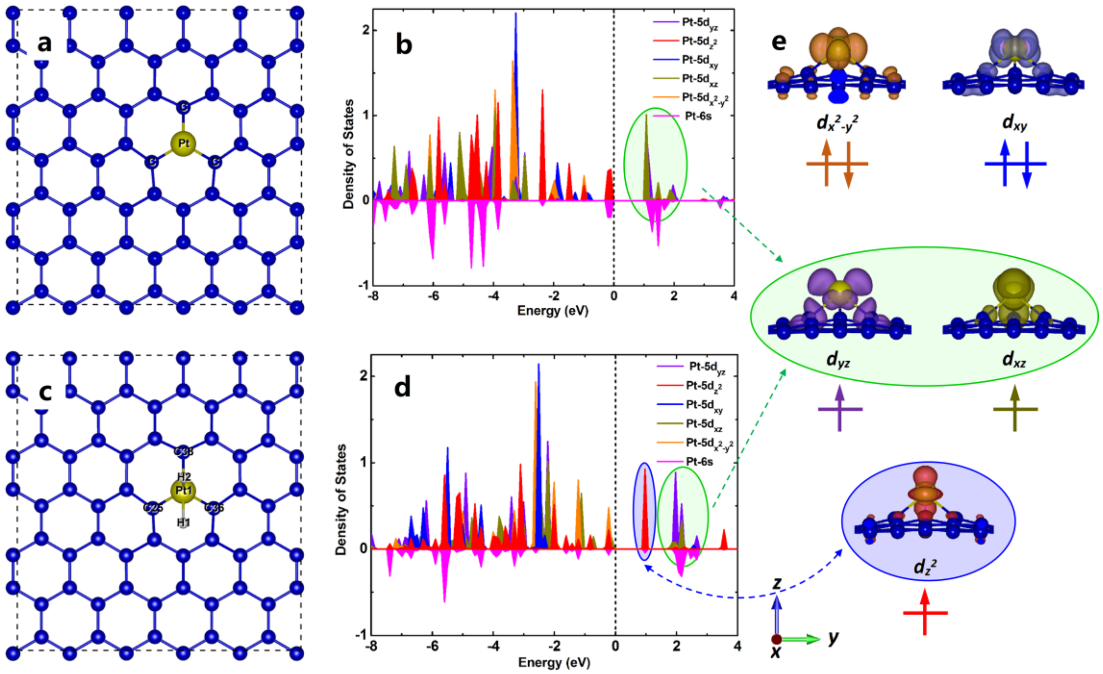

Supplementary Figure 30. (a), (c) The illustrated structure and (b), (d) partial density of states (PDOS) of the single Pt+SV on mesoporous carbon without and with H adsorption, respectively. The yellow, red and white balls indicate Pt, O, H, respectively. The positive y-axis shows the PDOS of Pt-d orbitals in Pt+SV-MC systems without and with H adsorption. The Fermi level is shifted to zero. (e) The suborbitals of the Pt-d near the Fermi level in Pt+SV-MC without and with H adsorption.

Supplementary Table 1. XAFS parameters of Pt single atoms materials, Pt/C and Pt foil. N, coordination number, R, distance between absorber and backscatter atoms.

**Pt-Pt**	**Shell**	**N**	**R (Å)**	**σ^2 (10^{-3}Å^2)**
**Pt_1/MC**	Pt-C/O	3.9	2.14	6.4
**Pt_1/TiO_2**	Pt-O	3.7	2.05	6.5
**Pt foil**	Pt-Pt	12	2.76	4.8

Supplementary Table 2. The Bader charge of the single Pt atom in the form of Pt-H_2O and H-Pt-OH, respectively.

	Pt+63H_2O	H-Pt-OH+62H_2O
Pt	-0.094	+0.408
O (in OH)	-1.315	-1.170
H (in OH)	+0.659	+0.668
H	+0.656	-0.048

Supplementary Table 3. The Pt-s/d partial charge and Pt oxidation-states (OS) of Pt-H_2O and H-Pt-OH in water environment, respectively. The occupation numbers for full occupancy of Pt-d orbitals are shown in bold.

	Pt+63H_2O	H-Pt-OH+62H_2O
s	0.515	0.505
d_{xy}	**1.868**	**1.902**
d_{yz}	**1.910**	**1.893**
d_{xz}	**1.876**	1.809
$d_{x^2-y^2}$	**1.880**	1.225
d_{z^2}	1.377	1.796
Pt OS	Pt (0)	Pt (+1~+2)

Supplementary Table 4. Calculated Pt adsorption energies (E_a), average Bader charge of Pt and C_{Pt} (The carbon atoms which are connected to the single platinum), and the average Pt-C bond lengths (l_{Pt-C}) when the Pt adsorbed on the SV, DV and edge sites of graphene. For comparison, the Pt adsorption properties on the perfect graphene (PG) was also examined. A positive Bader charge indicates the loss of electrons, where a negative value denotes electrons were gained.

	E_a (eV)	Bader charge (e)		l_{Pt-C} (Å)
		**Pt**	C_{Pt}	
**PG**	1.772	+0.011	-0.025	2.100
**SV**	7.353	+0.270	-0.195	1.939
**DV**	6.648	+0.448	-0.088	1.980
**Edge**	7.461	+0.205	-0.126	1.987

Supplementary Table 5. HER activity for different single atoms catalysts and Pt-based catalysts in 0.5 M H_2SO_4. For the Pt-based catalysts, the loading is corresponding to the content of Pt loading, meanwhile, the loading of other catalysts is the whole quality of catalysts. It indicates superior HER activity for Pt_1/MC in our work with respect to previous catalysts. (*The loading of all Pt-based HER catalysts is referring to Pt element).

**Catalyst**	**Loading* (mg cm^{-2})**	**Tafel slope (mV dec^{-1})**	**Overpotential at 10 mA cm^{-2} (mV)**	**Overpotential at 100 mA cm^{-2} (mV)**	**Ref.**
Pt_1/MC	0.010	26	25	65	This work
**Pt-MoS_2**	0.027	40	52	NA	8
**ALD50Pt+NGNs**	0.0076	29	~45	NA	9
**A-Ni-C**	0.283	41	34	112	10
**Co-NG**	0.285	82	147	NA	11
**Ni-doped graphene**	NA	45	~180	NA	12
**Pt+MoS_2**	0.018	96	12	NA	13
**Pt-CNSs/RGO**	0.078	29	~75	NA	14
**Pt/MoS_2/CC**	NA	49	18	~75	15
**Pt@Te-RGO/PI**	NA	55	100	NA	16
**PtNPS/CNFs**	0.0149	50	175	NA	17

Supplementary Table 6. Calculated Pt-C length (l_{Pt-C}), Pt-H length (l_{Pt-H}), Bader charge and magnetic moment of the Pt single atom adsorption single vacancy graphene with and without H chemisorption.

	l_{Pt-C} (Å)	l_{Pt-H} (Å)	Bader charge (e)		μ
			Pt	H	
**H-chemisorption**	1.976	1.682	+0.377	-0.239	1
**non-H**	1.939	-	+0.270	-	0

Supplementary Table 7. The size of Pt nanoparticles on the substrates prepared by the different approaches, as reported in the literature.

Sample	Precursor	Concentration	Structure	References
Ru-Pt core-shell	K_2PtCl_4	5 mM	Nanoparticles (~4 nm)	18
Pt@SiO_2	K_2PtCl_4	1.06 mM	core–shell structured Pt@mSiO_2 nanoparticles	19
Pt_xNi_{1-x}	K_2PtCl_4	4 mM	Pt-rich facets of 2–4 atomic layers in thickness	20
TMV-Pt	K_2PtCl_4	1 mM	Nanoparticles (~10 nm)	21
Pt/GC	K_2PtCl_4	0.026 mM	Nanoparticles (~2 nm)	22
THH Pt NCs	K_2PtCl_6	2 mM	Pt tetrahexahedral nanocrystals (53-144 nm)	23
Pt-Pd-rGO	H_2PtCl_6	1.93 mM	Pt-Pd-rGO nanoparticles (~10 nm)	24
Pt monolayer/metal NF/Ni foam	K_2PtCl_4	3 mM	Pt monolayer and multilayer films	25

Supplementary Table 8. The final atomic structures of Pt grown on several Pt-based catalysts with the corresponding Pt loadings.

Sample	Pt loading (wt %)	Structure	Ref.
Pt–MoS_2	1.7	Single atoms	26
Sample A (Pt_1/FeO_x)	0.17	Single atoms	27
Sample B (Pt/FeO_x)	2.5	Mixture of single atoms, two-dimensional Pt rafts consisting of fewer than 10 Pt atoms and three dimensional Pt clusters of size about 1 nm or less	27
Pt@MCM-22	0.11	Single atoms and a few large Pt clusters (about 0.7-1.0nm)	28
Pt-CN	0.16	Single atoms	29
Pt-CN	0.38	Single atoms and the sub-nanometer clusters	29
Pt/TiN	0.35	Single atoms	30
Pt/TiN	2	Single-atom Pt and very small Pt nanoparticles	30
Pt/CeO_2-polyhedra	1	Single atoms	31
Pt/WO_x	2.59	Single atom without obvious clusters	32
ALD50Pt/NGNs	2.5	individual Pt atoms and very small Pt clusters	33
Pt/ZTC	5.0 ± 0.2	Pt clusters about 4 nm	34
Pt/LSC	5.0 ± 0.2	1-2 nm Pt clusters	34
Pt/HSC	5.0 ± 0.2	Single atoms with no Pt cluster	34

Supplementary Table 9. The Bader charge, s/d-orbital occupation numbers and oxidation-states (OS) of the non-hydrogen and H-adsorption Pt single atoms which are loaded on the typical single vacancy and double vacancy MC systems without and with hydrogen adsorption, respectively. For example, Pt+SV+2H represent the Pt single atoms on SV with two hydrogen adsorption. The occupation numbers for full occupancy of Pt-d orbitals are shown in bold.

	Pt (Bader charge)	s	Occupation numbers (d-orbitals)					OS	Pt-d electrons
Pt+SV	+0.269	0.447	**1.863**	**1.865**	**1.864**	1.365	1.367	Pt (+1)	8.324
Pt+SV+2H	+ 0.391	0.470	**1.806**	**1.821**	1.450	1.430	1.448	Pt (+2)	7.955
Pt+DV	+0.348	0.488	**1.787**	**1.767**	**1.827**	1.487	1.426	Pt (+1)	8.294
Pt+DV+2H	+0.424	0.453	**1.803**	**1.701**	1.555	1.423	1.470	Pt (+2)	7.952

Supplementary References

1. Jones, J. *et al.* Thermally stable single-atom platinum-on-ceria catalysts via atom trapping. *Science* **353,** 150-154 (2016).
2. Qiao, B. *et al.* Single-atom catalysis of CO oxidation using Pt_1/FeOx. *Nat. Chem.* **3,** 634-641 (2011).
3. Conway, B. E., & Tilak, B. V. Interfacial processes involving electrocatalytic evolution and oxidation of H_2, and the role of chemisorbed H. *Electrochim. Acta* **47,** 3571-3594 (2002).
4. Parsons, R. The rate of electrolytic hydrogen evolution and the heat of adsorption of hydrogen. *Trans. Faraday Soc.* **54,** 1053-1063 (1958).
5. Nørskov, J. K. *et al.* Trends in the exchange current for hydrogen evolution. *J. Electrochem. Soc.* **152,** J23-J26 (2005).
6. Jiao, Y., Zhang, M., & Jaroniec, Z. S. Design of electrocatalysts for oxygen-and hydrogen-involving energy conversion reactions. *Chem. Soc. Rev.* **44,** 2060-2086 (2015).
7. Bligaard, K. *et al.* Trends in the exchange current for hydrogen evolution. *J. Electrochem. Soc.* **152,** J23-J26 (2005).
8. Xu, G. R., Hui, J. J., Huang, T., Chen, Y., & Lee, J. M. Platinum nanocuboids supported on reduced graphene oxide as efficient electrocatalyst for the hydrogen evolution reaction. *J. Power Sources* **285,** 393-399 (2015).
9. Cheng, N. *et al.* Platinum single-atom and cluster catalysis of the hydrogen evolution reaction. *Nat. Commun.* **7,** 13638 (2016).
10. Fan, L. *et al.* Atomically isolated nickel species anchored on graphitized carbon for efficient hydrogen evolution electrocatalysis. *Nat. Commun.* **7,** 10667 (2016).
11. Fei, H. *et al.* Atomic cobalt on nitrogen-doped graphene for hydrogen generation *Nat. Commun.* **6,** 8668 (2015).
12. Qiu H. J. *et al.* Nanoporous Graphene with Single-Atom Nickel Dopants: An Efficient and Stable Catalyst for Electrochemical Hydrogen Production. *Angew. Chem. Int. Ed.* **54,** 14031 (2015).
13. Huang, X. *et al.* Solution-phase epitaxial growth of noble metal nanostructures on dispersible single-layer molybdenum disulfide nanosheets. *Nat. Commun.* **4,** 1444 (2013).
14. Deng. J. *et al.* Triggering the electrocatalytic hydrogen evolution activity of the inert two-dimensional MoS_2 surface via single-atom metal doping. *Energy & Environ. Sci.* **8,** 1594-1601 (2015).
15. Luo, Y. *et al.* MoS_2 nanosheet decorated with trace loads of Pt as highly active electrocatalyst for hydrogen evolution reaction. *Electrochim. Acta* **219,** 187-193 (2016).
16. Zhang, A. *et al.* A novel Pt@Te-reduced graphene oxide/polyimide composite catalyst for hydrogen evolution. *Int. J. Hydrogen Energy.* **40,** 16238-16247 (2015).

17. Yang, T., Du, M., Zhu, H., Zhang, M., & Zou, M. Immobilization of Pt nanoparticles in carbon nanofibers: bifunctional catalyst for hydrogen evolution and electrochemical sensor. *Electrochim. Acta* **167,** 48-54 (2015).
18. Alayoglu, S. *et al.* Ru–Pt core–shell nanoparticles for preferential oxidation of carbon monoxide in hydrogen. *Nat. Mater.* **7,** 333-338 (2008).
19. Joo, S. H. *et al.* Thermally stable Pt/mesoporous silica core-shell nanocatalysts for high-temperature reactions. *Nat. Mater.* **8,** 126-131 (2009).
20. Cui, C. *et al.* Compositional segregation in shaped Pt alloy nanoparticles and their structural behaviour during electrocatalysis. *Nat. Mater.* **12,** 765-771 (2013).
21. Tseng, R. J. *et al.* Digital memory device based on tobacco mosaic virus conjugated with nanoparticles. *Nat. Nanotech.* **1,** 72-77 (2006).
22. Maillard, F. *et al.* Influence of particle agglomeration on the catalytic activity of carbon-supported Pt nanoparticles in CO monolayer oxidation. *Phys. Chem. Chem. Phys.* **7,** 385-393 (2005).
23. Tian, N. *et al.* Synthesis of tetrahexahedral platinum nanocrystals with high-index facets and high electro-oxidation activity. *Science* **316,** 732-735 (2007)
24. Bai, S. *et al.* Surface polarization matters: enhancing the hydrogen-evolution reaction by shrinking Pt shells in Pt-Pd-graphene stack structures. *Angew. Chem. Int. Ed.* **53,** 12120-12124 (2014).
25. Li, M. *et al.* Pt monolayer coating on complex network substrate with high catalytic activity for the hydrogen evolution reaction. *Sci. Adv.* **1,** e1400268 (2015).
26. Deng, J. *et al.* Triggering the electrocatalytic hydrogen evolution activity of the inert two-dimensional MoS_2 surface via single-atom metal doping. *Energy Environ. Sci.* **8,** 1594-1601 (2015).
27. Qiao, B. *et al.* Single-atom catalysis of CO oxidation using Pt_1/FeOx. *Nat. Chem.* **3,** 634-641 (2011).
28. Liu, L. *et al.* Generation of subnanometric platinum with high stability during transformation of a 2D zeolite into 3D. *Nat. Mater.* **16,** 132-138 (2017)
29. Li, X. *et al.* Single-atom Pt as co-Catalyst for enhanced photocatalytic H_2 evolution. *Adv. Mater.* **28,** 2427-2431 (2016).
30. Yang, S. *et al.* Single-atom catalyst of platinum supported on titanium nitride for selective electrochemical reactions. *Angew. Chem. Int. Ed.* **55,** 2058-2062 (2016)
31. Jones, J. *et al.* Thermally stable single-atom platinum-on-ceria catalysts via atom trapping. *Science* **353,** 150-154 (2016).
32. Wang, J. *et al.* Hydrogenolysis of glycerol to 1,3-propanediol under low hydrogen pressure over WO_x-supported single/pseudosingle atom Pt catalyst. *ChemSusChem.* **9,** 784-790 (2016).
33. Cheng, N. *et al.* Platinum single-atom and cluster catalysis of the hydrogen evolution reaction. *Nat. Commun.* **7,** 13638 (2016).
34. Choi, C. *et al.* Tuning selectivity of electrochemical reactions by atomically

dispersed platinum catalyst. *Nat. Commun.* **7,** 10922 (2016).
